# The solute carrier SPNS2 recruits PI(4,5)P_2_ to synergistically regulate transport of sphingosine-1-phosphate

**DOI:** 10.1016/j.molcel.2023.06.033

**Published:** 2023-08-03

**Authors:** Haiping Tang, Huanyu Li, Dheeraj Prakaash, Conrado Pedebos, Xingyu Qiu, David B. Sauer, Syma Khalid, Katharina Duerr, Carol V. Robinson

**Affiliations:** 1 Department of Chemistry, University of Oxford, Oxford OX1 3TA, UK; 2 Kavli Institute for Nanoscience Discovery, Oxford OX1 3QU, UK; 3 Centre for Medicines Discovery, Nuffield Department of Medicine, University of Oxford, Oxford OX3 7DQ, UK; 4 Department of Biochemistry, University of Oxford, Oxford OX1 3QU, UK; 5 OMass Therapeutics, Ltd., Oxford OX4 2GX, UK

**Keywords:** solute carriers, SLCs, SPNS2, sphingosine-1-phosphate, S1P, native MS, protein-lipid interactions, HDX-MS, molecular dynamics

## Abstract

Solute carrier spinster homolog 2 (SPNS2), one of only four known major facilitator superfamily (MFS) lysolipid transporters in humans, exports sphingosine-1-phosphate (S1P) across cell membranes. Here, we explore the synergistic effects of lipid binding and conformational dynamics on SPNS2’s transport mechanism. Using mass spectrometry, we discovered that SPNS2 interacts preferentially with PI(4,5)P_2_. Together with functional studies and molecular dynamics (MD) simulations, we identified potential PI(4,5)P_2_ binding sites. Mutagenesis of proposed lipid binding sites and inhibition of PI(4,5)P_2_ synthesis reduce S1P transport, whereas the absence of the N terminus renders the transporter essentially inactive. Probing the conformational dynamics of SPNS2, we show how synergistic binding of PI(4,5)P_2_ and S1P facilitates transport, increases dynamics of the extracellular gate, and stabilizes the intracellular gate. Given that SPNS2 transports a key signaling lipid, our results have implications for therapeutic targeting and also illustrate a regulatory mechanism for MFS transporters.

## Highlights

• SPNS2 interacts preferentially with PI(4,5)P_2_
• Mutation of potential PI(4,5)P_2_ binding sites inhibits SPNS2 transport activity
• PI(4,5)P_2_ synergistically enhances S1P-induced extracellular gate opening
• Lipid regulation of an MFS transporter prior to structure determination

## Graphical abstract

**Figure:**
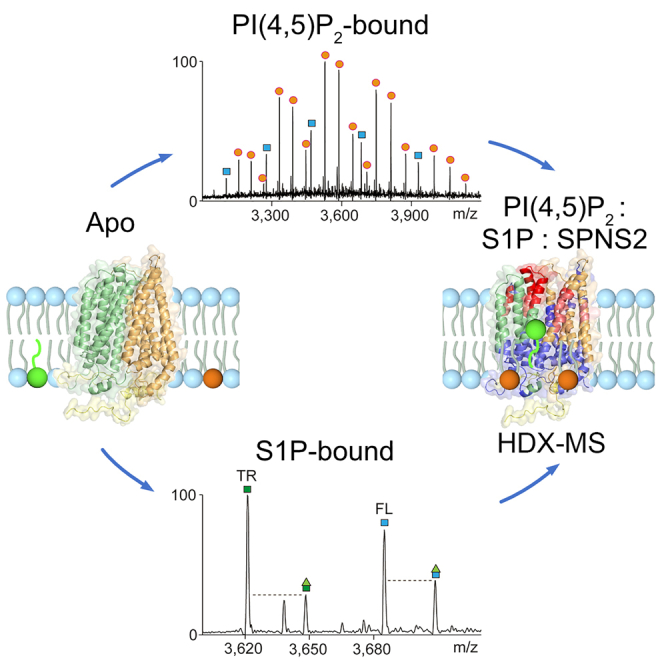

## Teaser

Tang et al. use native mass spectrometry to show that the lysolipid transporter SPNS2 binds preferentially to the phospholipid PI(4,5)P_2_. Using hydrogen-deuterium exchange to probe the dynamics of SPNS2, they show how PI(4,5)P_2_ and the signaling lipid S1P act together to promote conformational changes that facilitate substrate transport.

## Introduction

Sphingosine-1-phosphate (S1P) is a signaling lipid involved in the regulation of lymphocyte trafficking,^1^ embryonic development,^2^ inflammation,^3^ and many other physiological processes. Dysregulation of S1P levels leads to a number of human diseases, including autoimmune diseases,^4^ neurological disorders,^5^ and cancer.^6^^,^^7^ S1P is synthesized in cells by sphingosine kinases 1 and 2, which phosphorylate sphingosine,^8^ and subsequently exported out of cells by the S1P transporters, spinster homolog 2 (SPNS2),^9^^,^^10^ and major facilitator superfamily (MFS) domain containing 2B (MFSD2B).^11^ After export, extracellular S1P is sensed by cells via five G-protein-coupled receptors, S1P receptors 1 to 5, to activate downstream signaling pathways.^12^^,^^13^

The role of SPNS2 in S1P delivery was first discovered in zebrafish, where mutations in SPNS2 induced abnormal heart development, known as cardia bifida, a phenotype that could be rescued by injecting exogenous S1P.^10^ Further *in vivo* evidence showed that SPNS2-deficient mice had a decreased level of S1P in the blood.^14^ Moreover, SPNS2 is also able to export several S1P analogs, including dihydro-S1P and the immunomodulator FTY720 phosphate (FTY720-P).^14^^,^^15^ FTY720 (fingolimod) is approved for the treatment of multiple sclerosis, the most common autoimmune disorder of the central nervous system.^16^ Administered as a prodrug, FTY720 is phosphorylated by sphingosine kinase 2 in cells and exported to the extracellular space via SPNS2.^17^ This extracellular FTY720-P mimics S1P, interacting with S1P receptors and inducing them to internalize from cell membranes, thereby inhibiting pathogenic lymphocyte trafficking.^18^ In addition, deletion of SPNS2 prevented disease development in a mouse model of multiple sclerosis,^19^ indicating that SPNS2 is a promising new drug target for treating multiple sclerosis.

No structures for full-length human SPNS2 have been reported to date. A structure has been reported for an inward-facing conformation of a bacterial homolog of SPNS2^20^ and a structural model of human SPNS2 is available in the AlphaFold2 database (https://alphafold.ebi.ac.uk/).^21^^,^^22^ In this prediction, similar to the structure of the bacterial homolog, human SPNS2 has a canonical MFS fold that comprises two domains, the N domain and the C domain, with each domain consisting of six consecutive transmembrane helices (TMs). In the classic MFS transport model, these two domains use a rocker-switch mechanism to provide alternate access to substrate on each side of the membrane and thereby allow its translocation.^23^ In the AlphaFold2 model, TMs 1, 4, 7, and 10 line the central cavity of SPNS2 and, by analogy, constitute the transport pathway.^24^ TMs 2, 5, 8, and 11 form the interface between the N and C domains. TMs 3, 6, 9, and 12 are assembled on the periphery and maintain structural integrity.^24^ In addition to the core MFS fold, the predicted model of human SPNS2 has an additional putative N-terminal helix containing six arginine residues (Arg23–28), which are conserved among mammals (Figure S1A) and connected by a large disordered loop. This N-terminal region is designated as “low confidence” in the model from the AlphaFold2 database (Figure S1B). The significance of this region in terms of its structure and influence on function is challenging to discern.

Solute carriers (SLCs) are embedded in lipid bilayers and undergo significant conformational changes during their substrate transport cycle. Therefore, the surrounding lipids might be expected to play substantial roles in regulating transport function,^25^^,^^26^ oligomeric state,^27^ stability,^28^^,^^29^ and conformational dynamics.^30^^,^^31^ Noteworthy, in the case of membrane proteins, AlphaFold2 is not able to consider specific lipid binding that may induce secondary structure in intrinsically disordered regions.^21^ Although the majority of structural studies to date have focused on the effect of lipids on bacterial transporters,^25^^,^^32^^,^^33^^,^^34^ the modulation of mammalian SLCs by specific lipids on a lipid transporter has yet to be explored. Native mass spectrometry (native MS) is one of the few biophysical approaches capable of capturing lipid interactions and reporting on the time-resolved conformational dynamics associated with lipid binding. In such experiments, lipids are added to proteo-micelle solutions, and equilibrium dissociation constants (*K*_D_) are determined from peak intensities of the individual lipid-bound species. Here, we use this approach to discern lipid preferences for two SPNS2 variants, supporting our findings with functional studies. We then validate the AlphaFold2 model with results from hydrogen-deuterium exchange mass spectrometry (HDX-MS) for both proteins and use this model to assess the impact of lipid and substrate binding on the conformational dynamics of the protein. Supported by molecular dynamics (MD) simulations, we propose a mechanism for the initial recruitment and docking of PI(4,5)P_2_ by SPNS2 and the subsequent opening of the extracellular gate. Together, these approaches allow us to uncover the synergistic effects of biologically active lipids and a lipid substrate on SPNS2 transport activity.

## Results

### SPNS2 interacts preferentially with PI(4,5)P_2_

**Figure 1:**
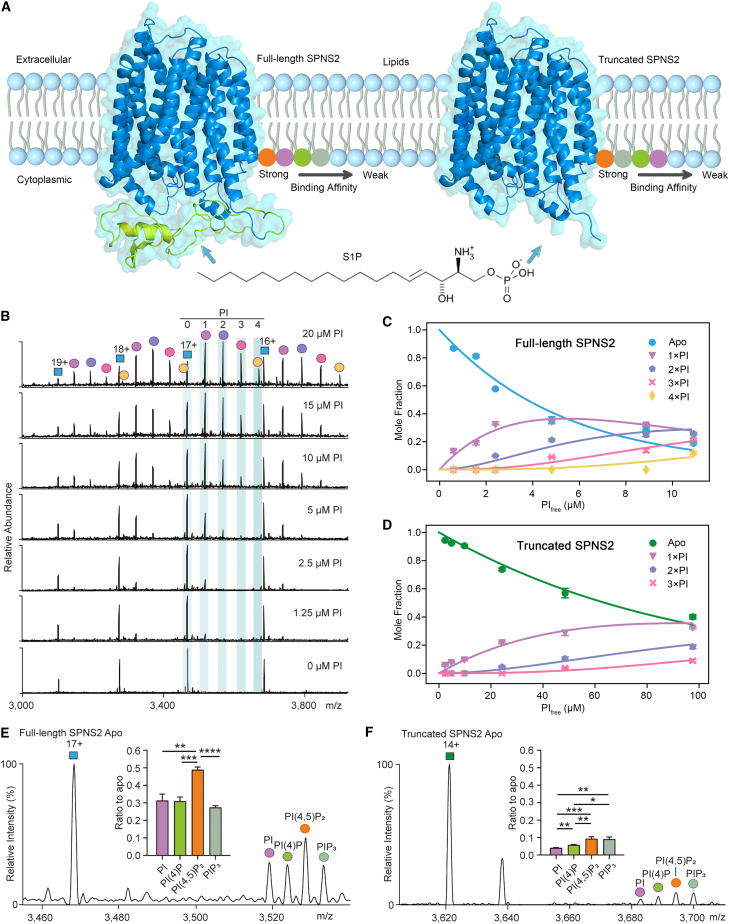
A predicted model of SPNS2 and the binding preference of PI derivatives to full-length and truncated SPNS2 (A) The AlphaFold2 predicted model of SPNS2 (blue) with the N terminus of the full-length protein (green) shown schematically, embedded within a phospholipid bilayer. PI derivatives: PI, PI(4)P, PI(4,5)P_2_, and PIP_3_ are shown as purple, green, orange, and gray spheres, respectively, with binding preference to full-length and truncated SPNS2 arranged via proximity to the transporter (according to the K_D_ values in Table S1). The structure of S1P is also shown. (B) Mass spectra recorded for delipidated full-length SPNS2 (5 μM) with increasing concentrations from 0 to 20 μM of PI (18:1/18:1). (C) Plot of the mole fraction of full-length SPNS2 and PI binding calculated from titration of PI (18:1/18:1) with a resulting fit (R2 = 0.97) from a sequential ligand-binding model (solid lines). Data are plotted as mean ± standard deviation (SD) (n = 3). (D) Plot of mole fraction of truncated SPNS2 with PI-bound states calculated from titration of PI(18:1/18:1) with a resulting fit (R2 = 0.99) from a sequential ligand-binding model (solid lines). Data are plotted as mean ± SD (n = 3). (E) A single charge state (17+) of full-length SPNS2 following incubation with an equimolar solution containing PI, PI(4)P, PI(4,5)P_2_, and PIP_3_ confirms preferred binding of PI(4,5)P_2_. Data are plotted as mean ± SD (n = 3). ^∗∗^p < 0.01. ^∗∗∗^p < 0.001. ^∗∗∗∗^p < 0.0001. (F) A single charge state (14+) of truncated SPNS2 following incubation with an equimolar solution containing PI, PI(4)P, PI(4,5)P_2_, and PIP_3_. Data are plotted as mean ± SD (n = 3). ^∗^p < 0.05. ^∗∗^p < 0.01. ^∗∗∗^p < 0.001.

Considering first the expression of SPNS2, we examined the AlphaFold2 model, which displays a typical MFS architecture but has an N-terminal region with a low-confidence helix and loop predicted^21^ (Figure S1B). Due to the unusual location of the AlphaFold2 model of the predicted N-terminal helix relative to other MFS transporters and to remove any preconception about the structure or location of the N terminus, we used steered MD simulations to obtain a starting model where the N terminus was outside the protein cavity (Figure 1A). We then expressed the full-length and truncated proteins (without the N-terminal 89 amino acids) in Expi293F cells using the BacMam system. Both proteins were purified in n-dodecyl-β-D-maltopyranoside (DDM) and had similar size exclusion chromatography profiles, purities, and thermostabilities (Figures S1C–S1F).

Both proteins were then subjected to native MS under the same experimental conditions. Well-resolved charge states were obtained for both full-length and truncated SPNS2, with adduct peaks tentatively assigned to lipids (∼863 Da) (Figures S2A and S2B). Combining these native MS results with lipidomics, we identified these adducts as phosphatidylinositols (PI) (PI [18:1/18:1] and PI [18:0/18:1]) (Figures S2C and S2D). Deconvolution of the mass spectra showed that the anticipated masses for full-length and truncated SPNS2 (Figures S2A and S2B) with additional proteomics identified an N-terminal acetylation (Figures S2E and S2F), a widespread protein translational modification among eukaryotes.^35^

To confirm our lipid identification, we first removed endogenous lipids^36^ then incubated delipidated SPNS2 with PI (18:1/18:1). The same adduct peaks as for the endogenously bound lipids (Figure 1B) confirm that full-length SPNS2 binds to PI. We then measured the *K*_D_ for individual PI binding events, observing a concomitant increase in the intensity of the PI-bound species as the concentration increased (Figure 1B). At PI concentrations >10 μM, PI-bound full-length SPNS2 predominated over unbound forms. At least four binding events were observed when PI concentrations were >20 μM (Figure 1B). Mole fractions of *apo* and lipid-bound species were calculated from the intensity of the peaks and fitted with a sequential ligand-binding model to determine the *K*_D_ for individual lipid binding events^37^ (Figure 1C; Table S1). We performed analogous *K*_D_ measurements for truncated SPNS2 and found that the binding affinity of PI to truncated SPNS2 is dramatically weaker (Figure 1D; Table S1). Together, these results confirm the high affinity of full-length SPNS2 for PI and imply a role for the protein’s N terminus in lipid binding.

We next examined the selectivity of full-length SPNS2 for other lipids, including phosphatidic acid (PA), phosphatidylserine (PS), phosphatidylglycerol (PG), phosphatidylethanolamine (PE), and phosphatidylcholine (PC). We measured *K*_D_s for all the above mentioned glycerophospholipids with 1-palmitoyl-2-oleoyl (16:0-18:1) acyl chains (Figure S3; Table S2). Interestingly, although PE and PC are the two most abundant glycerophospholipids found in mammalian cells, no apparent binding was observed for PC to full-length SPNS2, whereas *K*_D1_ for PE is 20-fold weaker than PI (Table S2). Notably, PI had the strongest binding affinity among these glycerophospholipid species, indicating that full-length SPNS2 has a strong preference for the inositol head group. The propensity for PI lipids to be phosphorylated *in vivo* prompted us to further investigate selectivity for different PI phosphates (PIPs). A competitive binding with equimolar ratios of PI, PI(4)P, PI(4,5)P_2_, and PIP_3_ (all 18:1/18:1) showed that peaks assigned to PI, PI(4)P, and PIP_3_ binding have similar intensities, but that the PI(4,5)P_2_ bound peak has the highest intensity (Figure 1E), consistent with its preferential interaction with full-length SPNS2.

### The N terminus of SPNS2 affects both PI(4,5)P_2_ and S1P binding

**Figure 2:**
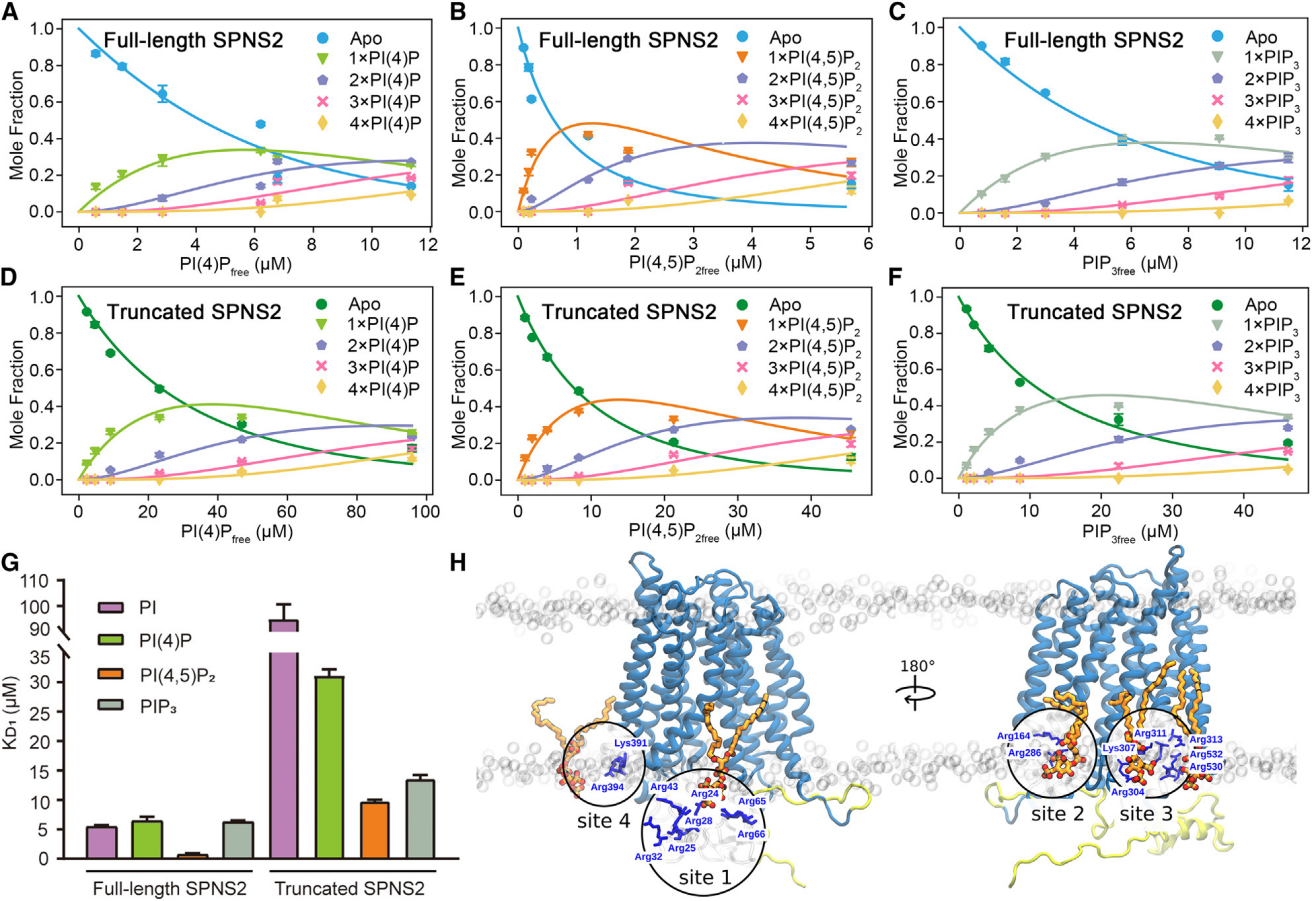
Determination of binding affinities for PI derivatives to full-length and truncated SPNS2 and potential PI(4,5)P_2_ binding sites on full-length SPNS2 (A–F) Binding curves for PI(4)P, PI(4,5)P_2_, and PIP_3_ binding to full-length SPNS2 (A, B, and C, respectively) and to truncated SPNS2 (D, E, and F, respectively). Data are plotted as mean ± SD (n = 3). (G) *K*_D1_ values for PI, PI(4)P, PI(4,5)P_2_, and PIP_3_ binding to full-length and truncated SPNS2. Data are plotted as mean ± SD (n = 3). (H) MD simulation reveals PI(4,5)P_2_ molecule (orange sticks) bound to the N terminus (binding site 1), and further three PI(4,5)P_2_ binding sites are shown (sites 2–4). SPNS2 is shown in blue (with the N terminus colored in yellow), the potential PI(4,5)P_2_ interacting residues are shown as blue sticks, and phosphorus atoms in the headgroups of membrane lipids are shown as gray spheres.

To understand further the role of the N terminus on PIP binding, we performed analogous binding experiments for N-terminally truncated SPNS2. Compared with the full-length protein, N-terminally truncated SPNS2 shows significantly reduced binding ratios for all investigated PI derivatives (Figure 1F). Comparing *K*_D_ values of PIPs, as before, for both full-length and N-terminally truncated SPNS2, we observed up to four PIP binding events with higher affinity for full-length SPNS2 than the N-terminally truncated protein (Figures 2A–2G; Table S1). Notably, PI(4,5)P_2_ has the highest affinity to both full-length and truncated SPNS2 with *K*_D1_ for the first PI(4,5)P_2_ binding of 0.7 ± 0.2 and 9.9 ± 0.1 μM, respectively (Table S1). Intriguingly, PI(4)P and PIP_3_ have binding affinities comparable to PI for full-length SPNS2 with a *K*_D1_ of ∼6 μM (Table S1), despite the fact that PI(4)P and PIP_3_ contain more phosphate groups than PI, indicating that binding is not merely an effect of increased charge. Unlike full-length SPNS2, truncated SPNS2 shows a significantly weaker affinity for all the PIPs (Figure 2G). Deletion of the N terminus caused more than 10-fold weaker affinity for PI(4,5)P_2_ compared with full-length SPNS2, indicating that the N terminus of SPNS2 is important not only for high-affinity PIP binding but also for dictating its preference for PI(4,5)P_2_.

Next, we carried out atomistic simulations of the AlphaFold2 SPNS2 model with the unstructured N terminus outside the protein cavity in a phospholipid bilayer containing PI(4,5)P_2_. PI(4,5)P_2_ bound to the N terminus (binding site 1), interacting with the Arginine-rich patch of Arg 23–28, Arg43, Arg65, and Arg66 (Figures 2H and S4). In addition, we found further PI(4,5)P_2_ binding sites on the periphery of the MFS domain bound primarily to the cytoplasmic loops TM2-3 (binding site 2), TM8-9 (binding site 4), and the intracellular helices 1 and 2 (ICH1 and ICH2, binding site 3) (Figures 2H and S4).

**Figure 3:**
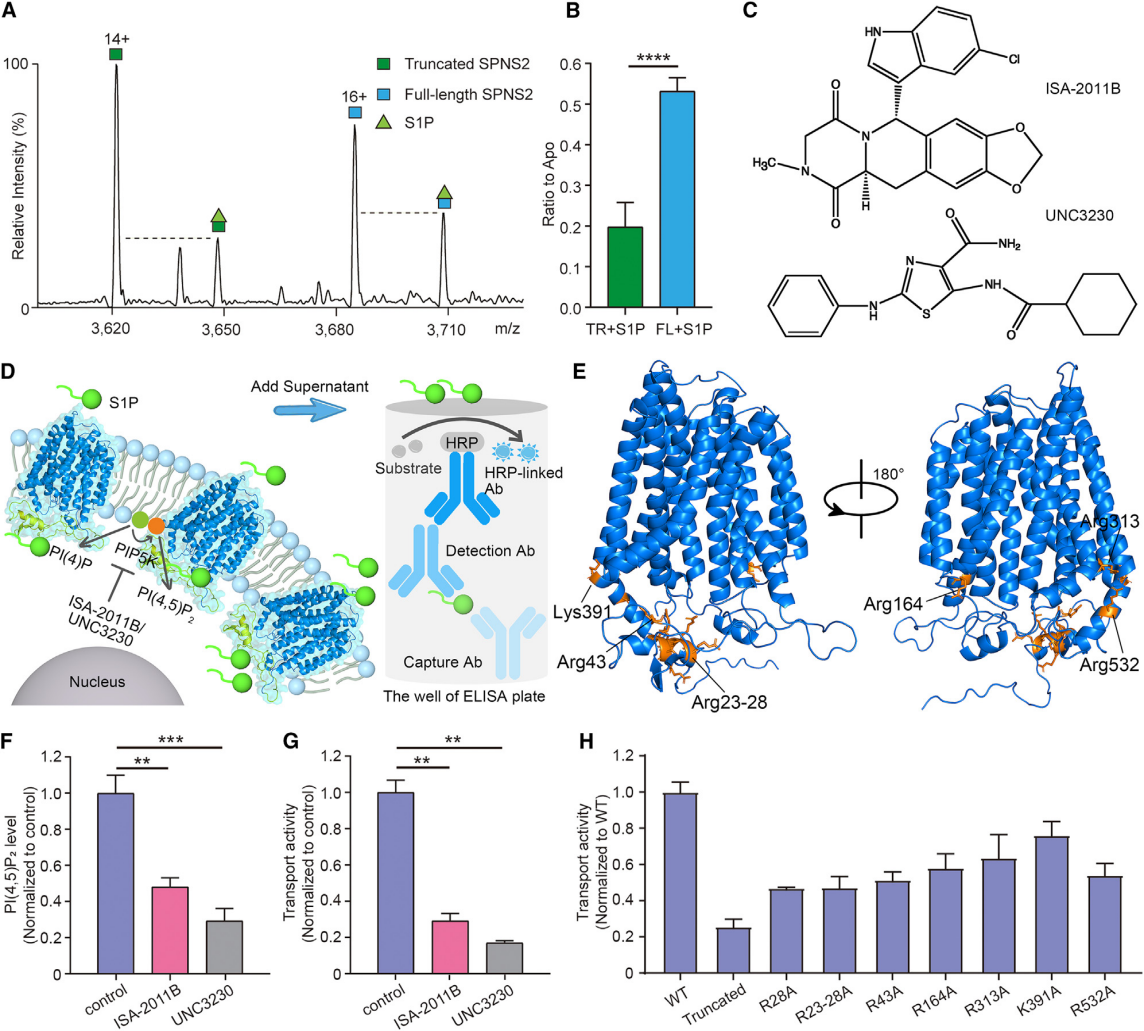
The effect of PI(4,5)P_2_ on S1P transport (A) Mass spectrum of equimolar ratios of full-length and truncated SPNS2 following incubation with substrate, S1P. (B) Relative ratios of S1P-bound peaks to the respective *apo* peaks confirm that full-length SPNS2 has a higher affinity for S1P. Data are plotted as mean ± SD (n = 3). ^∗∗∗∗^p < 0.0001. (C) Structures of the two PIP5K inhibitors ISA-2011B and UNC3230 used in the cell-based transport assay to inhibit synthesis of PI(4,5)P_2_. (D) Schematic illustration of the cell-based functional assay. S1P is synthesized in cells and exported across cell membranes by SPNS2. Extracellular S1P is detected by an enzyme-linked immunosorbent assay (ELISA). (E) Potential PI(4,5)P_2_ interacting residues corresponding to binding sites 1–4 are highlighted in the full-length SPNS2 model (orange). (F) PI(4,5)P_2_ levels of full-length SPNS2 overexpressing HEK293T cells treated with and without PIP5K inhibitors ISA-2011B and UNC3230. Data are plotted as mean ± SD (n = 3). ^∗∗^p < 0.01. ^∗∗∗^p < 0.001. (G) Extracellular S1P levels of full-length SPNS2 overexpressing HEK293T cells treated with and without PIP5K inhibitors ISA-2011B and UNC3230. Data are plotted as mean ± SD (n = 3). ^∗∗^p < 0.01. (H) Transport activity of full-length SPNS2 variants with point mutations at PI(4,5)P_2_ binding sites. WT, wild-type SPNS2. Truncated, N-terminal truncated SPNS2. The residues R23-R28 are mutated into alanines (R23-28A). Transport activities are normalized to FLAG expression (Figure S5B) and represented as a ratio to the wild type. Data are plotted as mean ± SD (n = 3).

We then explored whether the N terminus has an effect on substrate binding by adding S1P to an equimolar solution of full-length and truncated SPNS2. The resulting mass spectrum showed peaks corresponding to the S1P-bound full-length and truncated SPNS2 (Figure 3A). Plotting the intensity of peaks corresponding to S1P-bound states relative to those of the respective *apo* proteins revealed that S1P bound with higher affinity to full-length SPNS2 than the truncated variant (Figure 3B). Taken together, these results allow us to propose that the N terminus of SPNS2 has an effect on both PI(4,5)P_2_ and S1P binding. We hypothesize that PI(4,5)P_2_ and S1P act synergistically to facilitate S1P transport.

### PI(4,5)P_2_ plays a crucial role in S1P transport

To better understand the impact of PI(4,5)P_2_ on SPNS2 activity and to test our hypothesis, we established a cell-based assay for S1P transport by SPNS2 (Figure 3D). Using the full-length SPNS2 overexpressing cells as an assay platform, we subsequently sought to understand the role of PI(4,5)P_2_ in S1P transport by inhibiting PI(4,5)P_2_ production by two different PI 4-phosphate 5-kinases (PIP5Ks). After treating the SPNS2 overexpressing cells for 6 h with ISA-2011B^38^ and UNC3230,^39^ which are distinct chemical scaffolds (Figure 3C) and inhibit distinct PIP5K enzymes, we observed substantially reduced PI(4,5)P_2_ levels in both cases, with UNC3230 being the more effective inhibitor of PI(4,5)P_2_ production (Figure 3F).

Having established methods to reduce PI(4,5)P_2_ production, we then measured S1P secretion in the presence of each PIP5K inhibitor separately. Both treatments had no effect on SPNS2 overexpression levels (Figure S5A) and showed a decrease in S1P secretions from SPNS2 overexpressing cells (Figure 3G). Interestingly, the decrease in S1P secretion was more pronounced in the presence of the more effective inhibitor of PI(4,5)P_2_ production (UNC3230) (Figures 3F and 3G). The results from this functional assay therefore align with our proposal that levels of PI(4,5)P_2_ are directly related to the efficiency of S1P transport by SPNS2.

Next, we set out to confirm the importance of each MD-identified PI(4,5)P_2_ binding site by measuring the transport activity of mutants at each location. The arginine patch (Arg23–28) and six charged residues from the PI(4,5)P_2_ binding sites 1 to 4 were selected and individually mutated to alanine (Figure 3E). While confirming all the variants localized to the plasma membrane (Figure S5), we noted that mutation of any of the PI(4,5)P_2_ binding sites reduced S1P secretion (Figure 3H). In addition, the N-terminal truncated variant displayed the most impaired transport activity (Figure 3H). Together, these results reinforce the important roles of PI(4,5)P_2_ binding to the protein and the N terminus in S1P transport.

### Substrate S1P induces opening of the extracellular gate of SPNS2

Because our results indicate that PI(4,5)P_2_ and the N terminus regulate S1P transport by functional assays, we next sought to investigate the molecular details by examining the conformational dynamics of SPNS2. After optimizing the experimental conditions for time-resolved HDX-MS experiments of SPNS2 proteins, we detected 165 peptides with a sequence coverage of 90.8% for full-length SPNS2 and 109 peptides with a sequence coverage of 73% for truncated SPNS2 (Figure S6). Because the AlphaFold2 model has not been experimentally validated, we first used HDX-MS to assess the proposed elements of secondary structure in solution. All 12 predicted TMs, of both full-length and truncated SPNS2 proteins, show substantial protection from hydrogen exchange with low relative fractional uptake (RFU) (Figures S7A and S7B), indicating their structural integrity. The two ICH regions, the unstructured N- and C-terminal tails, and the interhelical regions show markedly less protection with high RFU, as expected for an MFS-fold protein.

**Figure 4:**
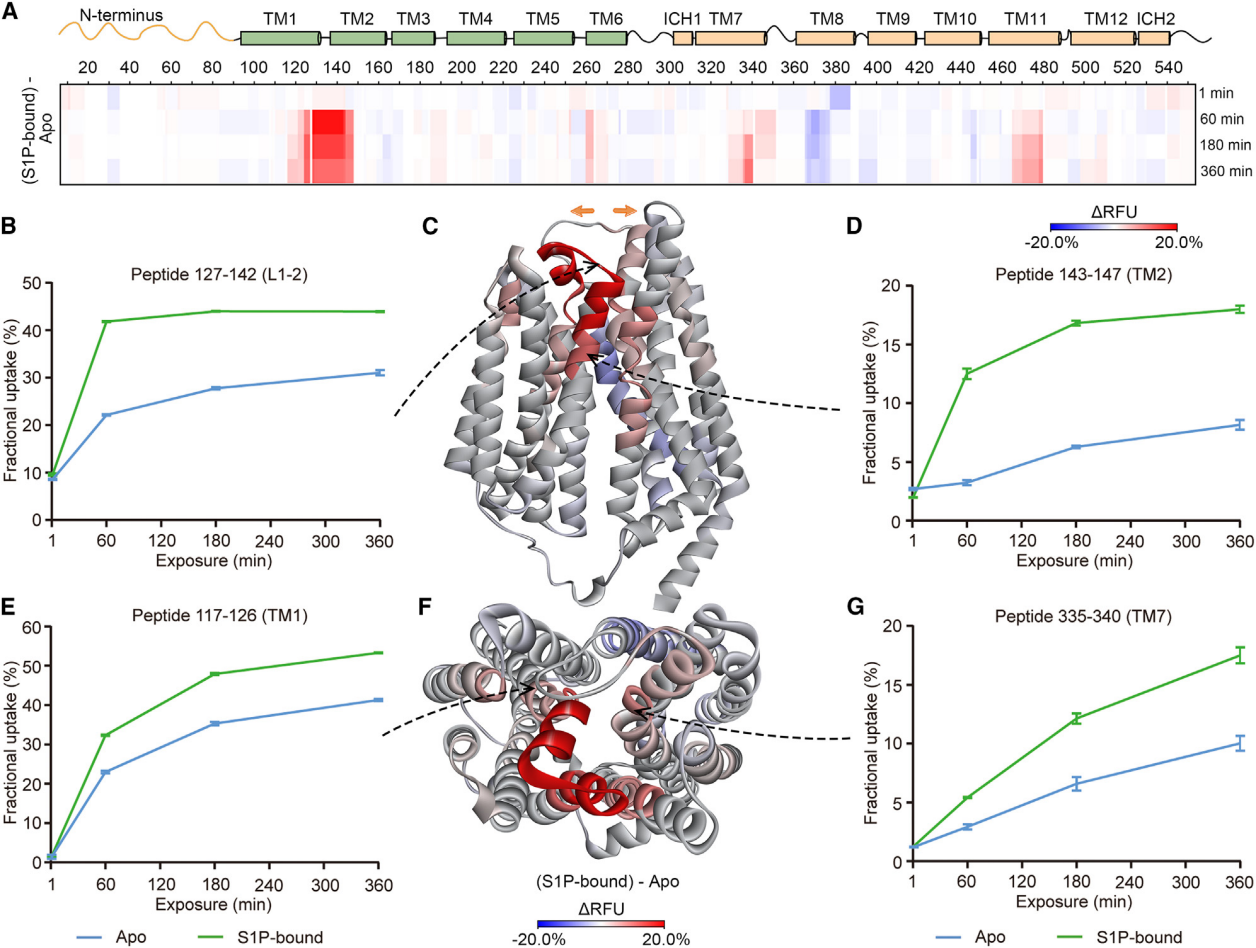
Conformational dynamics of S1P-bound SPNS2 (A) Heatmap comparing S1P-bound SPNS2 with *apo* SPNS2 via ΔRFU as a function of time. (B) Deuterium uptake plot for peptide 127–142. Data are plotted as mean ± SD (n = 3). (C) ΔRFU of S1P-bound SPNS2 compared with *apo* SPNS2 mapped onto the predicted model of SPNS2. Ribbon representation is viewed from membrane. (D) Deuterium uptake plot for peptide 143–147. Data are plotted as mean ± SD (n = 3). (E) Deuterium uptake plot for peptide 117–126. Data are plotted as mean ± SD (n = 3). (F) ΔRFU of S1P-bound SPNS2 compared with *apo* SPNS2 mapped onto the predicted model of SPNS2. Ribbon representation is viewed from extracellular side. (G) Deuterium uptake plot for peptide 335–340. Data are plotted as mean ± SD (n = 3).

Next, we performed HDX-MS of both SPNS2 proteins in the presence and absence of substrate S1P (Figures S7A and S7B). Comparing the HDX-MS results of SPNS2, with and without S1P, we found that S1P initially induces a rapid (<1 min) decrease in deuterium uptake at TM8 (Figure 4A). This result implies that S1P potentially enters the transporter from the membrane through a TM5–TM8 lateral opening. After 60 min or more, we observed that peptides from TM1, TM2, and TM7 displayed a substantial positive differential RFU (ΔRFU) in the presence of S1P (red shading in Figures 4A and S7C). Specifically, peptides 117–126, 127–142, 143–147, and 335–340 uptake more deuterium in the S1P-bound SPNS2 compared with the *apo* form (Figures 4B–4G). We then mapped the ΔRFU of SPNS2, with and without S1P, onto the proposed model of SPNS2. We found that peptides 117–126, 127–142, 143–147, and 335–340 are located at the extracellular ends of TM1, the loop L1-2, TM2, and TM7, respectively (Figures 4C and 4F). Similarly, we observed analogous conformational changes for truncated SPNS2 in the presence of S1P (Figure S7C). These observations are consistent with a dynamic opening of the extracellular gate of SPNS2 (the extracellular ends of TM1, the loop L1-2, TM2, and TM7) upon S1P binding.

### PI(4,5)P_2_ binding triggers allosteric regulation of conformational changes of SPNS2

**Figure 5:**
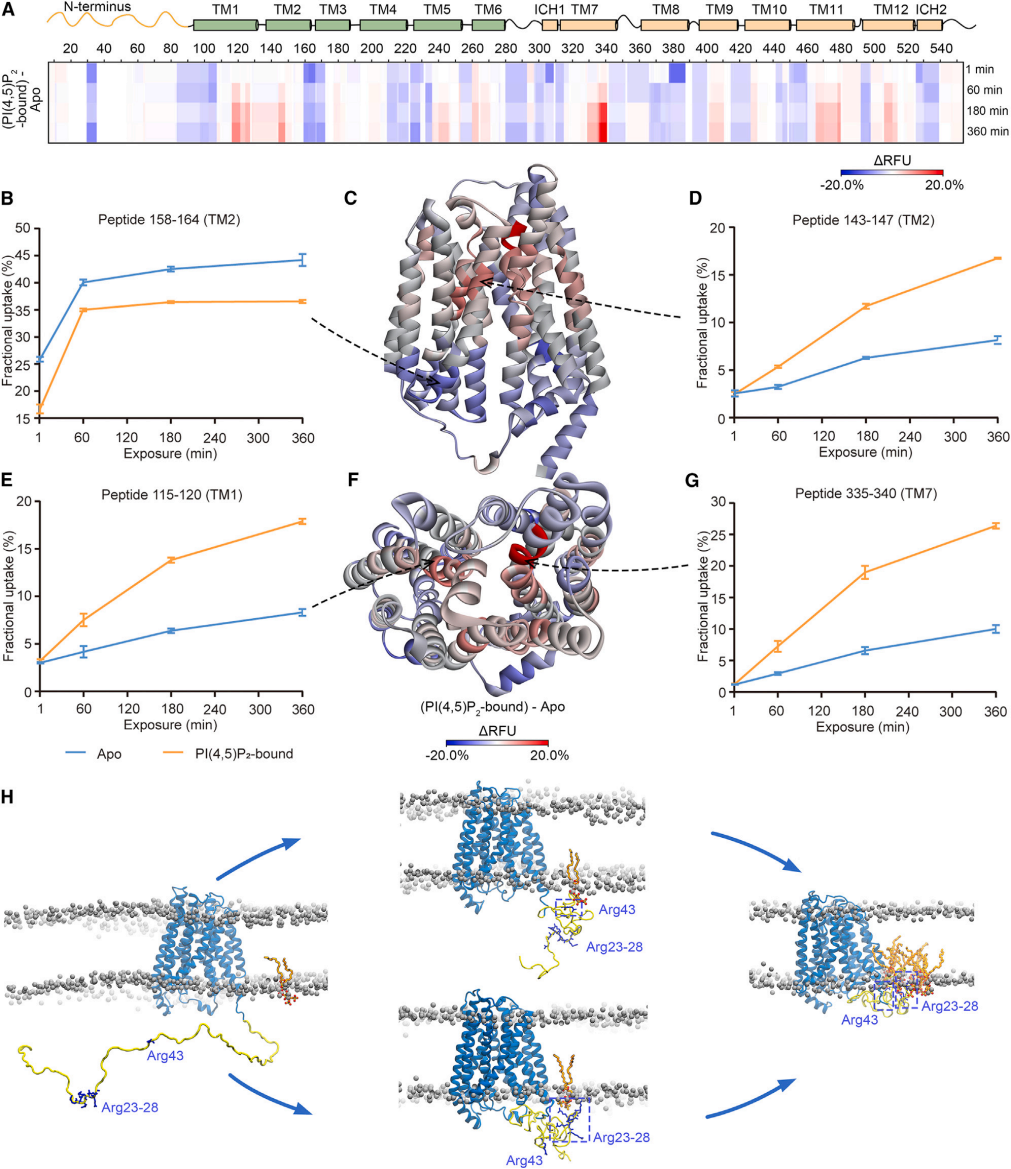
Conformational dynamics of PI(4,5)P_2_-bound SPNS2 and coarse-grained MD simulations of PI(4,5)P_2_ recruitment to SPNS2 (A) Heatmap representing ΔRFU, comparing PI(4,5)P_2_-bound SPNS2 with *apo* as a function of time. (B) Deuterium uptake plot for peptide 158–164. Data are plotted as mean ± SD (n = 3). (C) ΔRFU of PI(4,5)P_2_-bound SPNS2 compared with *apo* SPNS2 mapped onto the predicted model of SPNS2. Ribbon representation is viewed from membrane. (D) Deuterium uptake plot for peptide 143–147. Data are plotted as mean ± SD (n = 3). (E) Deuterium uptake plot for peptide 115–120. Data are plotted as mean ± SD (n = 3). (F) ΔRFU of PI(4,5)P_2_-bound SPNS2 compared with *apo* SPNS2 mapped onto the predicted model of SPNS2. Ribbon representation is viewed from extracellular side. (G) Deuterium uptake plot for peptide 335–340. Data are plotted as mean ± SD (n = 3). (H) Snapshots from CG simulations of SPNS2 in a PI(4,5)P_2_ containing phospholipid bilayer (back-mapped to atomistic resolution). Starting from the initial state, Arg43 or the arginine patch (Arg 23–28) interacts with PI(4,5)P_2_ molecule, and the N terminus guides the PI(4,5)P_2_ molecule closer to SPNS2. SPNS2 is shown in blue (with the N terminus colored in yellow), phospholipids are shown on a white surface (with the phosphates shown as gray spheres), and PI(4,5)P_2_ is shown as orange sticks.

To test the effect of PI(4,5)P_2_ on the conformational dynamics of full-length and truncated SPNS2, we carried out analogous HDX experiments to those described above but in the presence and absence of PI(4,5)P_2_ (Figure S7). By calculating the ΔRFU of full-length and truncated SPNS2, with and without PI(4,5)P_2_, we observe that PI(4,5)P_2_ causes a rapid (<1 min) overall decrease in deuterium uptake, notably at the N terminus, as well as TM1, L2-3, L4-5, ICH1, TM8, L10-11, and ICH2 (Figures 5A and S7C). Subsequently (>60 min), we observe an increase in deuteration on TM1, TM2, TM7 (most notably), and TM11 (Figures 5A and S7C). Overlaying the ΔRFU on the model of SPNS2, we found that in the presence of PI(4,5)P_2_ decreased deuterium uptake occurs mainly at the N terminus and the cytoplasmic side of SPNS2 (see, for example, peptide 158–164 from TM2 (Figures 5B and 5C). Meanwhile, we observed increased deuteration at peptides 143–147 from the extracellular side of TM2 (Figure 5D), indicative of an allosteric regulation of TM2 by PI(4,5)P_2_. Analogous allosteric regulation is also found for TM1 and TM7 (Figures 5E–5G). In addition, peptides 28–34 from the N terminus are protected from exchange rapidly (<1 min) in the presence of PI(4,5)P_2_ (Figure 5A), consistent with our proposal of PI(4,5)P_2_ binding in this region. Together, these data indicate that PI(4,5)P_2_ binds on the intracellular side of SPNS2, inducing extensive protection beyond that which could be attributed to individual PI(4,5)P_2_ binding sites, to allosterically induce conformational changes on the extracellular side (Figure 5C).

To investigate how PI(4,5)P_2_ induces conformational changes to the N terminus, we performed coarse-grained (CG) simulations of the full-length SPNS2 in a phospholipid bilayer containing PI(4,5)P_2_. We found that the first 89 residues at the N terminus adopted a range of different conformations over the course of the simulation. Although PI(4,5)P_2_ is initially randomly placed in the inner leaflet of the bilayer (Figure 5H), the positively charged guanidinium group of residues of Arg43 or the Arg patch (Arg 23–28) gradually extends to interact with PI(4,5)P_2_ (Figure 5H). Later in the trajectory, the N terminus guides the PI(4,5)P_2_ molecule closer to the SPNS2 protein (Figure 5H). Taken together, these results are consistent with the N terminus of SPNS2 participating in binding PI(4,5)P_2_ and imply the N terminus recruiting PI(4,5)P_2_ to the transporter.

### S1P-induced extracellular gate opening is enhanced synergistically by PI(4,5)P_2_

**Figure 6:**
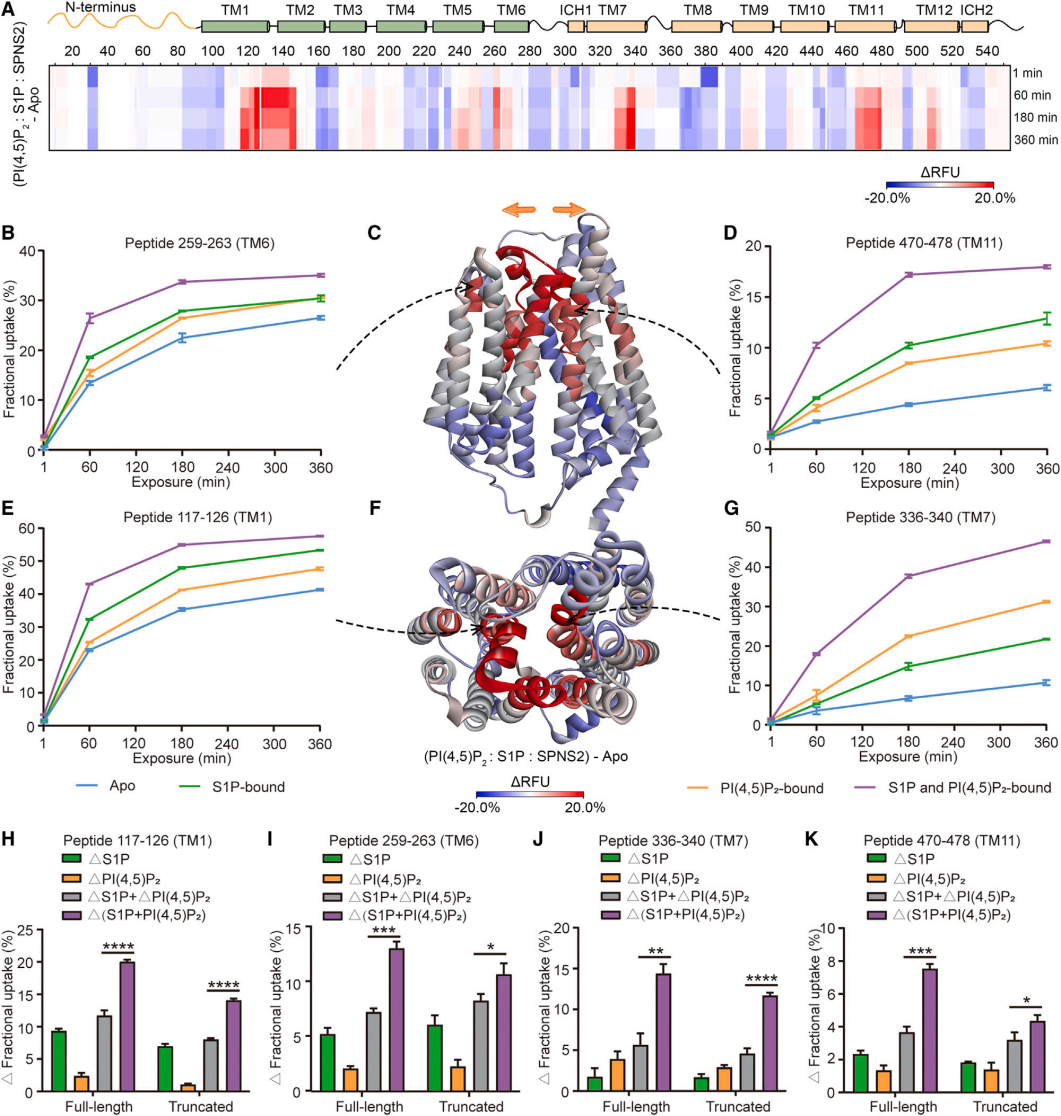
Conformational dynamics of SPNS2 in the ternary complex with both S1P and PI(4,5)P_2_. (A) Heatmap representing ΔRFU as a function of time, comparing PI(4,5)P_2_: S1P: SPNS2 with *apo* SPNS2. (B) Deuterium uptake plot for peptide 259–263. Data are plotted as mean ± SD (n = 3). (C) ΔRFU of PI(4,5)P_2_: S1P: SPNS2 compared with *apo* SPNS2 mapped onto the predicted model of SPNS2. Ribbon representation is viewed from membrane. (D) Deuterium uptake plot for peptide 470–478. Data are plotted as mean ± SD (n = 3). (E) Deuterium uptake plot for peptide 117–126. Data are plotted as mean ± SD (n = 3). (F) ΔRFU of PI(4,5)P_2_: S1P: SPNS2 compared with *apo* SPNS2 mapped onto the predicted model of SPNS2. Ribbon representation is viewed from extracellular side. (G) Deuterium uptake plot for peptide 336–340. Data are plotted as mean ± SD (n = 3). (H–K) Changes in fractional deuterium uptake of peptides 117–126 (H), 259–263 (I), 336–340 (J), and 470–478 (K) for full-length and truncated SPNS2 at 60 min labeling. All data are plotted as mean ± SD (n = 3). ^∗^p < 0.05. ^∗∗^p < 0.01. ^∗∗∗^p < 0.001. ^∗∗∗∗^p < 0.0001.

In order to characterize the combined effects of S1P and PI(4,5)P_2_ binding on the conformational dynamics of SPNS2, we performed HDX-MS experiments of S1P-bound SPNS2 in the presence and absence of PI(4,5)P_2_ (Figures S7A and S7B). When comparing the ternary complex (PI(4,5)P_2_: S1P: SPNS2) directly with the *apo* protein, we observed pronounced increases in deuterium uptake on the extracellular ends of TM1, TM2, TM6, TM7, TM11, and TM12 and a concomitant marked decrease at the N terminus and the cytoplasmic ends of helices TM1, L2-3, L4-5, ICH1, TM8, L10-11, and ICH2 (Figures 6A, 6C, and S7C). Comparing peptides from TM regions across the protein, specifically 117–126, 259–263, 336–340, and 470–478, greater deuteration was observed in the ternary complex (PI(4,5)P_2_: S1P: SPNS2) compared with *apo* or S1P-bound SPNS2 (Figures 6B–6G). Together, these results indicate that PI(4,5)P_2_ binding in the presence of substrate induces a larger opening of the extracellular gate than S1P alone.

To explore this proposed synergy, we compared the *apo* state RFU with full-length and truncated SPNS2 in complex with S1P (ΔS1P), PI(4,5)P_2_ (Δ PI(4,5)P_2_), and both S1P and PI(4,5)P_2_ present simultaneously (Δ(S1P + PI(4,5)P_2_)). We found that the increased deuterium uptake induced by both S1P and PI(4,5)P_2_ present simultaneously [Δ(S1P + PI(4,5)P_2_)] is larger than the summed results of S1P and PI(4,5)P_2_ individually (ΔS1P + ΔPI(4,5)P_2_) for peptides 117–126 (TM1), 259–263 (TM6), 336–340 (TM7), and 470–478 (TM11) of full-length SPNS2 (Figures 6H–6K). Similar synergistic effects were observed for the same regions of truncated SPNS2 (Figures 6H–6K). These HDX results demonstrate that PI(4,5)P_2_ and S1P act synergistically to induce conformational changes in SPNS2 to promote extracellular gate opening and facilitate S1P transport. Importantly, these results also indicate that the N terminus is not involved in the synergistic conformational changes induced by PI(4,5)P_2_ and S1P. Rather, when combined with our observations that truncated SPNS2 has lower affinity for PI(4,5)P_2_ and that S1P transport is significantly impaired, we deduce that the main function of the N terminus is the recruitment of PI(4,5)P_2_, which subsequently leads to more efficient S1P transport.

## Discussion

**Figure 7:**
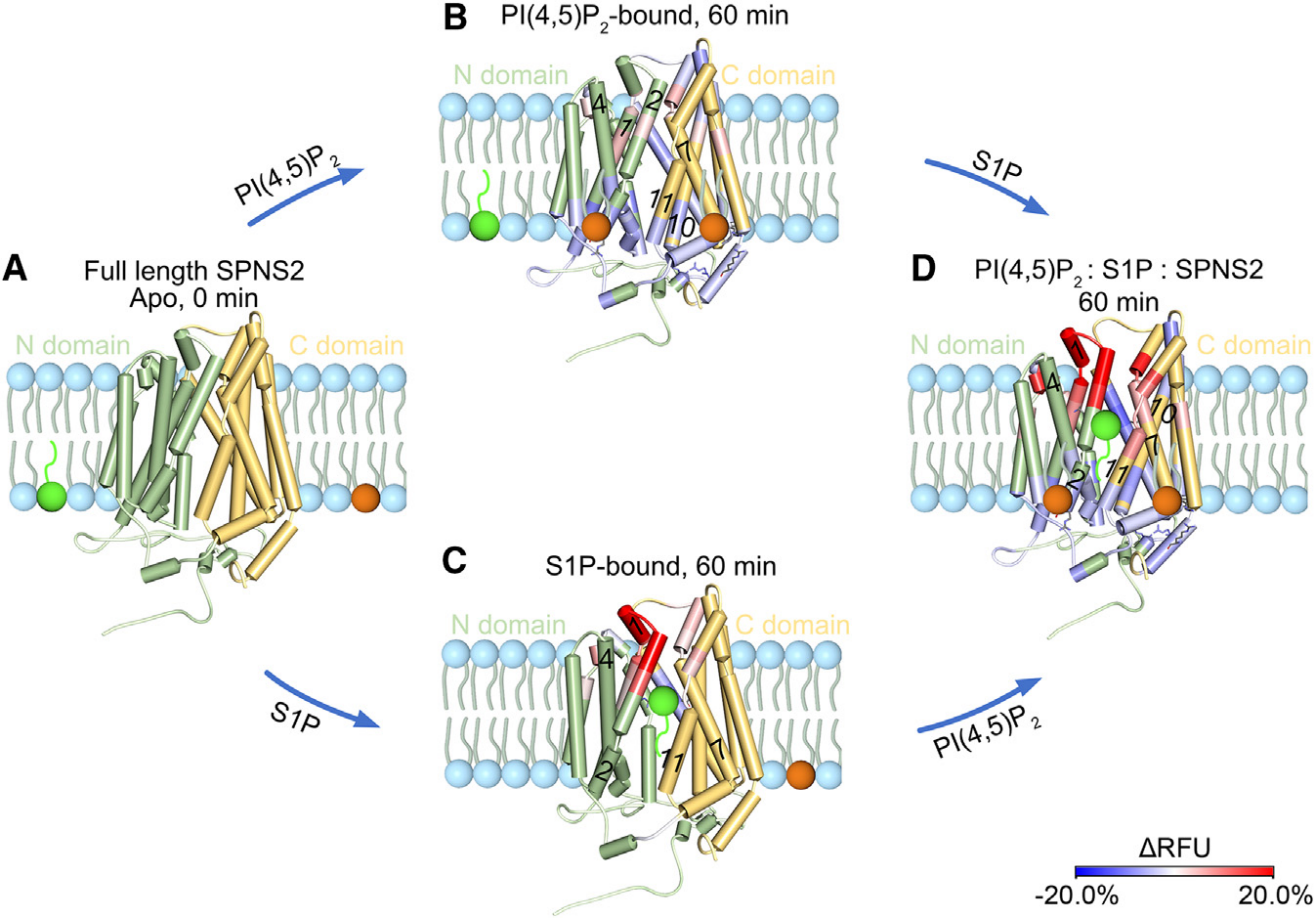
Proposed transport model based on time-resolved conformational changes of SPNS2 upon binding of PI(4,5)P_2_ and S1P All conformational changes are mapped onto the predicted model of SPNS2 and represent. (A) *apo* SPNS2 at 0 min. (B) ΔRFU of PI(4,5)P_2_-bound SPNS2 compared with *apo* SPNS2 at 60 min labeling. (C) ΔRFU of S1P-bound SPNS2 compared with *apo* SPNS2 at 60 min labeling. (D) ΔRFU of PI(4,5)P_2_: S1P: SPNS2 compared with *apo* SPNS2 at 60 min labeling. The helices are colored according to the corresponding HDX results above. N and C domains are shown in green and yellow, respectively, with S1P and PI(4,5)P_2_, green and orange spheres. A greater deuteration of the helices on the extracellular side indicates PI(4,5)P_2_ synergistically enhances S1P-induced opening of the extracellular gate while stabilizing the intracellular gate.

Here, we used native MS to compare individual lipid binding events with the S1P transporter SPNS2. We discovered a preference for PI(4,5)P_2_ binding to the full-length protein with preferential occupancy of up to four binding sites. In support of our functional studies, we found impaired S1P transport activity by SPNS2 upon inhibition of PI(4,5)P_2_ synthesis or mutations of PI(4,5)P_2_ binding sites. Through a series of comparative HDX experiments, we showed that PI(4,5)P_2_ and S1P work synergistically to trigger the necessary conformational changes for opening the extracellular gate while stabilizing the cytoplasmic face. These conformational changes are in accord with the current view of the rocker-switch mechanism proposed for MFS transporters and subsequently refined for the lysophosphatidylcholine transporter MFSD2A.^40^ Our data also identify important features of SPNS2 not previously reported for an MFS transporter, including the following: (1) The N terminus plays an important role in the recruitment and binding of PI(4,5)P_2_, (2) PI(4,5)P_2_ stabilizes the cytoplasmic face, and (3) the synergistic action of the substrate binding with PI(4,5)P_2_ induces further conformational changes to facilitate S1P transport. We summarize these findings and propose a lipid-mediated S1P translocation mechanism based on our time-resolved HDX data (Figure 7). Starting from the *apo* state (A), either PI(4,5)P_2_ is recruited to the inward-open SPNS2 and subsequently binds to the intracellular side of SPNS2 to stabilize the intracellular gate or S1P enters the central cavity to induce the opening of the extracellular gate (C). This extracellular gate opening is further augmented by PI(4,5)P_2_ binding. Although the order of lipid binding events would be difficult to define, it is clear from this study that the lipids act synergistically to stabilize a transition state, resulting in a larger opening of the extracellular gate to allow S1P release (D).

To date, mechanistic understanding of human SLCs has been hampered by the absence of high-resolution structures and the consideration of regions where secondary or tertiary structures are absent or dynamic. These regions are generally removed or difficult to resolve in high-resolution structure determination, and consequently, their critical roles are often overlooked. In this study, we identified regions of the S1P transporter SPNS2, some with weak secondary structure that are crucial in recruiting PI(4,5)P_2_ and reveal that this lipid allosterically enhances substrate-induced opening of the extracellular gate. The lipid recruitment and regulation mechanism may also be operative for conveying substrates in other MFS family members, as shown here for S1P in SPNS2. Importantly, because our work was achieved in advance of conventional structural information, it marks a step change in native MS. Until this point, native MS of membrane proteins has added detailed information to structures determined via either X-ray crystallography or cryo-EM but has not yet been used without the support of other structural biology approaches. Our study therefore showcases a new methodology to study the lipid regulation of an MFS transporter that not only provides mechanistic insight but also informs conditions for trapping distinct conformational states and enlightens optimal construct design for downstream structural studies.

Considering the implications for human health, multiple sclerosis is the most common autoimmune disorder of the central nervous system. Fingolimod (FTY720) has been approved for the treatment of multiple sclerosis; however, certain side effects involving bradycardia and macular edema have been observed.^41^ A more favorable approach for treating multiple sclerosis may be to interfere with the S1P signaling upstream of S1P receptors, for example by reducing S1P release through the inhibition of SPNS2 transport activity. Therefore, our results showing PI(4,5)P_2_ regulation of SPNS2 suggest a novel strategy for inhibiting SPNS2 by allosteric modulators. In addition, deletion of SPNS2 creates higher ratios of effector T cells and natural killer cells that can subsequently attack tumor cells and decrease metastatic burden.^42^ Therefore, in this case, inhibiting SPNS2 activity may prove a novel modality for immuno-oncology. Moreover, because impaired lipid synthesis will compromise PI(4,5)P_2_ levels, which themselves serve as effectors of multiple downstream proteins,^43^ mechanistic understanding of how S1P signaling is coordinated by PI(4,5)P_2_ has the potential to inform multifactorial interventions.

### Limitations of the study

Given the difficulties inherent to controlling the internal environment with the cell-based functional assay, we are unable to determine the driving force for SPNS2 in this study. One of the key challenges going forward is to develop further approaches to study native membrane proteins in more native-like bilayer environments. Although currently technically challenging, such an approach will enable links between naturally occurring post-translational modifications and interactions with regulatory lipids to be defined in native cellular environments.

## STAR★Methods

### Key resources table

**Table:** 

REAGENT or RESOURCE	SOURCE	IDENTIFIER
**Antibodies**		

Monoclonal Anti-FLAG M2 antibody	Sigma	Cat# F3165-.2MG
Anti-β-actin antibody	Cell Signaling Technology	Cat# 3700S

**Bacterial and virus strains**		

DH10Bac Competent Cells	Thermo Fisher Scientific	Cat# 10361012
Stellar Competent Cells	Takara	Cat# 636763

**Chemicals, peptides, and recombinant proteins**		

n-Dodecyl-b-D-maltopyranoside (DDM)	Anatrace	Cat# D310
18:1 PI	Avanti Polar Lipids	Cat# 850149P-500μg
18:1 PI(4)P	Avanti Polar Lipids	Cat# 850151P-500μg
18:1 PI(4,5)P_2_	Avanti Polar Lipids	Cat# 850155P-500μg
18:1 PI(3,4,5)P_3_	Avanti Polar Lipids	Cat# 850156P-500μg
16:0-18:1 PA	Avanti Polar Lipids	Cat# 840857P-500mg
16:0-18:1 PS	Avanti Polar Lipids	Cat# 840034P-500mg
16:0-18:1 PG	Avanti Polar Lipids	Cat# 840457P-500mg
16:0-18:1 PE	Avanti Polar Lipids	Cat# 850757P-500mg
16:0-18:1 PC	Avanti Polar Lipids	Cat# 850457P-500mg
Sphingosine 1-phosphate	Sigma	Cat# 73914-1MG
Deuterium oxide	Sigma	Cat# 151882-100G
Lipofectamine 2000	Thermo Fisher Scientific	Cat# 11668019
ISA-2011B	MedChemExpress	Cat# HY-16937-5mg
UNC3230	Cayman Chemical	Cat# 16401-5 mg-CAY

**Critical commercial assays**		

Human S1P ELISA kit	MyBioSource	Cat# MBS2516132
PI(4,5)P_2_ ELISA kit	Echelon Biosciences	Cat# K-4500

**Experimental models: Cell lines**		

Human: 293T cells	ATCC	Cat# CRL-3216
Human: Expi293F cells	Thermo Fisher Scientific	Cat# A14527
Insect: Sf9 cells	Thermo Fisher Scientific	Cat# 11496015

**Deposited data**		

Raw data	This paper	https://doi.org/10.17632/vcwttwj4z7.1

**Recombinant DNA**		

pcDNA5/TO empty vector	Thermo Fisher Scientific	Cat# V103320
pHTBV1.1 with C-terminal twin-Strep and 10×His tag	This paper	N/A
pHTBV1.1-SPNS2-GFP	This paper	N/A
pHTBV1.1-truncated SPNS2	This paper	N/A
pcDNA5/TO-SPNS2-FLAG	This paper	N/A
pcDNA5/TO-truncated SPNS2-FLAG	This paper	N/A
pcDNA5/TO-SPNS2(R23-28A)-FLAG	This paper	N/A
pcDNA5/TO-SPNS2(R28A)-FLAG	This paper	N/A
pcDNA5/TO-SPNS2(R43A)-FLAG	This paper	N/A
pcDNA5/TO-SPNS2(R164A)-FLAG	This paper	N/A
pcDNA5/TO-SPNS2(R313A)-FLAG	This paper	N/A
pcDNA5/TO-SPNS2(K391A)-FLAG	This paper	N/A
pcDNA5/TO-SPNS2(R532A)-FLAG	This paper	N/A
pcDNA5/TO-EGFP-SPNS2	This paper	N/A
pcDNA5/TO-EGFP-truncated SPNS2	This paper	N/A
pcDNA5/TO-EGFP-SPNS2(R23-28A)	This paper	N/A
pcDNA5/TO-EGFP-SPNS2(R28A)	This paper	N/A
pcDNA5/TO-EGFP-SPNS2(R43A)	This paper	N/A
pcDNA5/TO-EGFP-SPNS2(R164A)	This paper	N/A
pcDNA5/TO-EGFP-SPNS2(R313A)	This paper	N/A
pcDNA5/TO-EGFP-SPNS2(K391A)	This paper	N/A
pcDNA5/TO-EGFP-SPNS2(R532A)	This paper	N/A

**Software and algorithms**		

GraphPad Prism 9	GraphPad	https://www.graphpad.com/
Volocity 6.3.1	PerkinElmer	http://www.cellularimaging.com
Xcalibur 4.4	Thermo Fisher Scientific	https://thermo.flexnetoperations.com/
Proteome Discoverer 2.5	Thermo Fisher Scientific	https://thermo.flexnetoperations.com/
Lipid Search 4.2	Thermo Fisher Scientific	https://thermo.flexnetoperations.com/
MassLynx V4.1	Waters	N/A
ProteinLynx Global Server 2.5.1	Waters	N/A
DynamX 3.0	Waters	N/A

**Other**		

Superdex 200 Increase 10/300 GL column	GE Healthcare	Cat# 28-9909-44
Acclaim PepMap 100, C18, 75 μm × 15 cm	Thermo Fisher Scientific	Cat# 164568
Enzymate BEH Pepsin Column 2.1 × 30 mm	Waters	Cat# 186007233
ACQUITY UPLC BEH C18 1.7 μm 1.0x100 mm	Waters	Cat# 186002346

### Resource availability

#### Lead contact

Further information and requests for resources and reagents should be directed to and will be fulfilled by the lead contact, Carol V. Robinson (carol.robinson@chem.ox.ac.uk).

#### Materials availability

Plasmids generated in this study are available from the lead contact upon request. Materials that can be shared via a Material Transfer Agreement.

### Experimental model and study participant details

#### Cell culture

*E. coli* cells were cultured in LB medium (Sigma) at 37 °C. The *E. coli* strain Stellar cells were used to generate and amplify plasmids for SPNS2 mutations and strain DH10Bac cells were used to generate bacmids.

Sf9 insect cells (Thermo Fisher Scientific) were cultured in Sf-900 II SFM medium (GIBCO) at 27 °C. The baculovirus were generated and amplified in Sf9 cells in Sf-900 II SFM medium supplemented with 2% (v/v) fetal bovine serum (FBS) (GIBCO).

Expi293F suspension cells were cultured in Freestyle 293 expression medium (GIBCO) and grown in an orbital shaker at 37 °C and 8% CO_2_.

HEK293T cells were cultured in Dulbecco’s Modified Eagle’s Medium (DMEM) (GIBCO) supplemented with 10% FBS and grown in an incubator at 37 °C and 5% CO_2_.

### Method details

#### Expression and purification of SPNS2

The full-length human SPNS2 gene, and a construct with the first 89 amino acids deleted, were cloned into plasmid pHTBV1.1 containing a C-terminal tobacco etch virus (TEV) protease cleavage site followed by twin-Strep and 10×His affinity tags. Baculoviruses for these constructs were generated according to the previously described protocols.^44^ Briefly, the plasmids were transformed into DH10Bac to generate recombinant bacmid DNA. The bacmid DNA was then transfected to insect Sf9 cells using GeneJuice Transfection Reagent (Sigma) to generate recombinant baculovirus according to manufacturer’s instruction. The baculoviruses were generated and amplified in Sf9 insect cells. The resulting baculovirus was harvested and used to infect Expi293F cells. 1 L of Expi293F cells cultured in Freestyle 293 expression medium (GIBCO) was infected with 3% baculovirus (v/v) in the presence of 0.5% (v/v) 1M sodium butyrate. Transfected cells were grown in an orbital shaker for 48 h at 37 °C, 8% CO_2_ and 75% humidity before being harvested. The cell pellets were resuspended to extraction buffer (300 mM NaCl, 50 mM HEPES pH 7.5, 1% DDM) in the presence of cOmplete Protease Inhibitor Cocktail tablets (Roche) and solubilized at 4 °C for 1 h with gentle rotation. The insoluble materials were pelleted at 51,000× g for 40 min. The supernatants were incubated with pre-equilibrated TALON resin (Takara) and allowed to bind for 1 h at 4 °C. The resin was poured on a gravity-flow column and washed with TALON wash buffer (300 mM NaCl, 50 mM HEPES pH 7.5, 10 mM imidazole, 10 mM MgCl_2_, 1 mM ATP and 0.03% DDM). Protein was eluted with TALON elution buffer (300 mM NaCl, 50 mM HEPES pH 7.5, 300 mM imidazole and 0.03% DDM). The elution solution containing SPNS2 was incubated with pre-equilibrated Strep-Tactin XT Superflow resin (IBA-Lifesciences) for 1 h at 4 °C. The resin was poured on a gravity-flow column and washed with Strep wash buffer (300 mM NaCl, 50 mM HEPES pH 7.5, 0.03% DDM). Protein was eluted with Strep elution buffer (300 mM NaCl, 50 mM HEPES pH 7.5, 30 mM D-biotin and 0.03% DDM) followed by tag-cleavage with TEV protease overnight and reverse IMAC purification using TALON resin. The tag cleaved SPNS2 proteins were concentrated using centrifugal concentrator with 100 kDa cut off (Sartorius) and subjected to size exclusion chromatography using a Superdex 200 10/300 GL column (GE Healthcare) pre-equilibrated with gel filtration buffer (150 mM NaCl, 20 mM HEPES pH 7.5, 0.02% DDM). Peak fractions were pooled and concentrated for subsequent experiments.

#### Thermal stability measurement by nanoscale differential scanning fluorimetry (nanoDSF)

The purified proteins were diluted to a final concentration of 5 μM in gel filtration buffer and loaded into Plex nanoDSF Grade High Sensitivity Capillaries (NanoTemper Technologies). Intrinsic protein fluorescence was measured through a temperature ramp 20 to 95 °C (1 °C/min) using a Prometheus NT.48 (NanoTemper Technologies). Melting temperatures were determined from the first derivative of the 330 nm to 350 nm fluorescence ratio.

#### Lipids and S1P preparation

All lipids used in this study were purchased from Avanti Polar Lipids and 2 mM stock solutions were prepared following a previously described method.^45^ In brief, dried lipid films were resuspended in MS buffer (200 mM ammonium acetate buffer pH 7.5, 0.017% DDM) and sonicated for 10 min before dilution. S1P powder was obtained from Sigma and dissolved in 0.3M NaOH to make the stock solution (25 mg/ml). The lipids and S1P were diluted into MS buffer (200 mM ammonium acetate buffer pH 7.5, 0.017% DDM) for native MS experiments.

#### Native MS experiments

Purified SPNS2 proteins were buffer exchanged into MS buffer (200 mM ammonium acetate buffer pH 7.5, 0.017% DDM) using Micro Bio-Spin P-6 columns according to manufacturer’s instruction, loaded into a gold coated needle prepared in-house and immediately subjected to native MS analysis on a Q Exactive UHMR Hybrid Quadrupole-Orbitrap Mass Spectrometer (Thermo Fisher Scientific) as previously described.^46^ The following parameters were used: capillary voltage was set to 1.2 kV, injection flatapole was set to 5 V, inter flatapole lens was at 4 V, bent flatapole at 2 V, source fragmentation was set to 50 and resolution was set to 17500 at m/z 200. Trapping gas pressure was set to 7.5. An optimized HCD energy (100 V) was applied to remove the detergent micelle from the proteins. The same conditions were used for full-length and truncated SPNS2 proteins. Data were analysed using Xcalibur 4.4.

#### Lipid binding experiments

SPNS2 proteins were delipidated to remove the endogenous bound lipids according to the protocol described previously.^36^ For lipid titrations, a final concentration of 5 μM SPNS2 proteins were mixed with various concentrations of lipid solutions and incubated on ice for 10 min before being injected in the mass spectrometer. The lipid bound mole fraction ratio was calculated by dividing the intensity of lipid bound peaks by the total intensity of all observed species. The mole fraction ratios were exported in tab delimited text format for subsequent processing with a previous described custom Python scripts to determine *K*_*D*_ values.^37^^,^^47^ Competitive binding assays were conducted by incubating the delipidated full-length and truncated SPNS2 with equimolar ratios of lipids on ice for 10 min before being injected in the mass spectrometer. S1P binding experiments were performed by incubating S1P with an equimolar mixture of full-length and truncated SPNS2 on ice for 10 min before native MS analysis.

#### Lipidomics analysis

The endogenous bound lipids from SPNS2 proteins were extracted by adding chloroform-methanol (2:1, v/v) as previously described.^48^ The extracted lipids were dried and resuspended in 80% methanol. For LC-MS/MS analysis, the lipids were separated on a C18 column (Acclaim PepMap 100, C18, 75 μm × 15 cm, Thermo Fisher Scientific) using a Dionex UltiMate 3000 RSLC nano LC System using a previously described mobile phase and gradient.^49^ The column eluent was loaded to Orbitrap Eclipse Tribrid mass spectrometer (Thermo Fisher Scientific). The Orbitrap Eclipse was operated in negative mode using data-dependent acquisition. The detailed mass spectrometer parameters are as follows: spray voltage, -1.9 kV; capillary temperature, 320°C; mass range, 250-1500; full MS resolution, 120K; MS/MS resolution, 15K; NCE, 25/30/35; cycle time, 1.5s. The data was analysed by LipidSearch 4.2 software (Thermo Fisher Scientific).

#### Proteomics analysis

The full-length and truncated SPNS2 proteins were separated by SDS-PAGE, respectively. The gel bands of interest were excised from the gel, reduced with 25 mM of TCEP and alkylated with 55 mM iodoacetamide. In-gel digestion was then carried out at 37 °C overnight with sequencing grade modified trypsin and chymotrypsin for full-length SPNS2 proteins and truncated SPNS2 respectively. The digested peptides were extracted twice with 0.1% trifluoroacetic acid in 50% acetonitrile aqueous solution for 30 min. Extracts were then centrifuged in a SpeedVac vacuum concentrator (Thermo Fisher Scientific) to reduce the sample volumes. For LC-MS/MS analysis, the peptides were separated on a C18 column (Acclaim PepMap 100, C18, 75 μm × 15 cm, Thermo Fisher Scientific) using a Dionex UltiMate 3000 RSLC nano LC System and subjected to Orbitrap Eclipse Tribrid mass spectrometer (Thermo Fisher Scientific). Mobile phase A consisted of water and 0.1% formic acid, and mobile phase B consisted of 80% acetonitrile and 0.1% formic acid. The Orbitrap Eclipse was operated in positive mode using data-dependent acquisition. The detailed mass spectrometer parameters are as follows: spray voltage, 2.0 kV; capillary temperature, 320°C; mass range, 375-1500; full MS resolution, 120K; MS/MS resolution, 30K; NCE, 30; cycle time, 3 s. The data was analysed by Proteome Discoverer 2.5 (Thermo Fisher Scientific).

#### Cell-based functional assay

The full length, truncated and mutated SPNS2 genes, with C-terminal flag tags, were cloned into the pcDNA5/TO plasmid. The plasmid was transfected into HEK 293T cells for 24 h via Lipofectamine 2000 (Thermo Fisher Scientific) according to the manufacturer’s protocol. The SPNS2 overexpressing cells were seeded at density of 1×10^6^ per well in a 6-well plate and were treated with 50 μM ISA-2011B (MedChemExpress) or UNC3230 (Cayman Chemical) for 6 h. After treatment, the cell media were harvested and centrifuged for 20 min at 1,000× g at 4°C and supernatant S1P was measured using a S1P ELISA kit (MyBioSource) and the cell pellets were collected for PI(4,5)P_2_ measurement, as described below. For the SPNS2 mutants all constructs were transfected into HEK 293T cells for 24 h via Lipofectamine 2000 (Thermo Fisher Scientific) and seeded at a density of 1×10^6^ per well. After 6 h, the cell media were harvested and used for S1P measurement using S1P ELISA kit (MyBioSource).

#### Cellular PI(4,5)P_2_ quantification

Each cell pellet was resuspended in 1 ml of ice-cold 0.5 M trichloroacetic acid (TCA), incubated on ice for 5 min and centrifuged at 3,000 rpm for 7 min at 4°C. The pellets were washed twice with 1 ml of 5% TCA /1 mM EDTA. Afterward, neutral lipids were extracted by adding 1 ml of MeOH: CHCl_3_ (2:1), vortexed for 10 min and centrifuged at 3,000 rpm for 5 min. The supernatants were discarded, and the extraction step was repeated. The acidic lipids were extracted by adding 0.75 ml MeOH: CHCl_3_:12 M HCl (80:40:1), vortexed for 25 min and centrifuged at 3,000 rpm for 5 min. The supernatants were transferred to a new 2 ml tube. 0.25 ml of CHCl_3_ and 0.45 ml of 0.1 M HCl were added to the supernatant, vortexed for 30 s and centrifuged at 3,000 rpm for 5 min to separate organic and aqueous phases. The organic phase was collected and dried by SpeedVac vacuum concentrator (Thermo Fisher Scientific). The samples were resuspended in PBS containing 0.25% protein stabilizer and sonicated for 10min before PI(4,5)P_2_ quantification using a PI(4,5)P_2_ ELISA kit (Echelon Biosciences).

#### Western blot

Cell pellets collected from cell-based functional assays were resuspended in lysis buffer (300 mM NaCl, 50 mM HEPES pH 7.5, 2% DDM) in the presence of cOmplete Protease Inhibitor Cocktail tablets (Roche). Proteins were separated by SDS-PAGE and transferred to PVDF membranes (Thermo Fisher Scientific). The membranes were blocked in blocking buffer (5% milk dissolved in PBS supplement with 0.1% Tween 20, PBST) for 1 h at room temperature and incubated with Anti-FLAG antibody (Sigma, 1:1000 dilution) or Anti-β-actin antibody (Cell Signaling Technology, 1:2000 dilution) in blocking buffer for 2 h at room temperature. The membranes were washed five times with TBST, each time for 5 min. Then the membranes were incubated with Anti-mouse IgG HRP-linked secondary antibody (Cell Signaling Technology, 1:1000 dilution) in blocking buffer for 1 h at room temperature. The membranes were washed five times with TBST and were developed using SuperSignal™ West Pico PLUS Chemiluminescent Substrate (Thermo Fisher Scientific). The images were acquired using Bio-Rad ChemiDoc XRS+ imaging system and analysed using Image Lab software (Bio-Rad).

#### Live cell imaging

The full length, truncated and mutated SPNS2 genes with N-terminal GFP fusion were cloned into pcDNA5/TO plasmid. The plasmid was transfected into HEK 293T cells for 24 h via lipofectamine 2000 (Thermo Fisher Scientific) according to the manufacturer’s protocol. Live cells were imaged with a Perkin Elmer Spinning Disk Microscope. 100×Olympus PlanApo Objective with Numerical Aperture of 1.4 was used. GFP was excited by the 488 nm laser and the fluorescence images were taken using EM-CCD camera (Hamamatsu). Images were analysed using Volocity 6.3.1 software (Perkin Elmer).

#### HDX-MS experiment

Before labelling, full-length and truncated SPNS2 proteins were diluted to 20 μM using equilibration buffer (20 mM HEPES pH 7.5, 150 mM NaCl and 0.02% DDM). PI(4,5)P_2_ and S1P were diluted from prepared stock solutions using equilibration buffer. In the case of S1P binding experiment, SPNS2 proteins were incubated with 10-fold molar excess S1P for 20 min. For PI(4,5)P_2_ binding experiment, SPNS2 proteins were incubated with 5-fold molar excess PI(4,5)P_2_ for 20 min. To study the synergistic binding of both lipid and substrate, SPNS2 was first incubated with a 10-fold molar excess S1P and subsequently 5-fold molar excess PI(4,5)P_2_ was added and equilibrated for 20 min. To initiate the HDX reaction, 5 μl of each sample was incubated with 50 μl of D_2_O buffer (20 mM HEPES pD 7.5, 150 mM NaCl and 0.02% DDM) for a time course of 1, 60, 180 and 360 min, then quenched by 55 μl of ice-cold quenching solution (100 mM HEPES pH 2.0, 150 mM NaCl, 0.02% DDM). 80 μl of quenched sample was loaded into nanoACQUITY UPLC System (Waters) and digested by Enzymate BEH Pepsin Column (2.1 × 30 mm, Waters) at 20°C. The digested peptides were separated by ACQUITY UPLC BEH C18 column (1.7 μm, 1.0 × 100 mm, Waters). Mass spectra were acquired in MS^E^ mode using Synapt G2-Si HDMS mass spectrometer (Waters). Peptides from un-deuterated samples were identified by ProteinLynx Global Server 2.5.1 (Waters). HDX data were analysed by DynamX 3.0 (Waters). RFU was calculated by dividing the measured deuterium uptake by the theoretically maximum deuterium uptake.

#### MD simulations

To construct a model for atomistic MD simulations, a predicted model of the SPNS2 was initially obtained from AlphaFold2.^21^ However, to obtain a starting model where the unstructured N-terminal domain was located outside the protein cavity and in the cytoplasmic region, atomistic steered MD simulations were performed on the AlphaFold2 model. This refined model with the N-terminus outside the protein was then submitted to the CHARMM-GUI server^50^^,^^51^ and the Membrane Builder tool^52^ was used to create a membrane with a lipid composition of DPPC:PI(4,5)P_2_ (90:10%). Systems were explicitly solvated with water, and ions were added to both neutralize the system with and to provide a salt concentration of 0.15 M of KCl.

The GROMACS simulation suite (2020.3)^53^ was used to perform MD simulations. Atomistic simulations used the CHARMM36m force field^54^^,^^55^ and the TIP3P water model.^56^ Our model was relaxed by performing energy minimization using the steepest descent algorithm followed by several steps of equilibration simulations during which positional restraints on the protein were gradually released. After the equilibration phase, twelve production MD simulations of 50 ns each (total of 600 ns per system) were performed at a temperature of 340 K. The higher temperature in simulations provides faster sampling of conformational transitions and lipid interactions. The final snapshot of the most representative outcome of these simulations was used as a starting structure in further simulations extended for another 250 ns in 3 replicate simulations (750 ns in total) at 340 K. Replicate simulations were performed with different starting velocities to ensure distinct trajectories, and to enhance conformational sampling. The velocity rescale thermostat^57^ with a time constant of 1 ps was used to achieve a constant temperature. The pressure was maintained at 1 bar using a semi-isotropic, Parrinello-Rahman barostat^58^ with a time constant of 1 ps. Covalent bonds were constrained using the P-LINCS algorithm.^59^ The Particle mesh Ewald method^60^ was used to treat long-range electrostatics, with a cut-off value set to 1.2 nm (the same as the van der Waals cut-off). The leap-frog integrator with a 2 fs time-step was employed, saving frames every 100 ps.

The Martini 2.2 force field was employed for all CG simulations.^61^ The martinize script was used to obtain a CG model of the AlphafFold2 predicted model, while the N-terminus disordered region was modelled as a stretched coil using the Modeller package to avoid initial protein-protein interaction bias. The insane script^62^ was used to embed the resultant CG protein in a membrane containing the same lipid composition as the atomistic simulations. The system was solvated using standard Martini water and counterions were added to both neutralize the charges and to provide an overall concentration of 0.15 M within the system. CGMD simulations were performed for 4 microseconds in 3 replicates (12 microseconds in total) using a 20 fs time-step and frames were saved every 500 ps.

Visual Molecular Dynamics^63^ and PyMOL^64^ were used for visualization and preparation of figures. PyLipID^64^^,^^65^ was employed to obtain the PI(4,5)P_2_ binding sites in the transporter and calculate their residence times. CG2AT2^66^ was used to backmap snapshots from the CG simulations to atomistic resolution in order to visualize the SPNS2 recruiting PI(4,5)P_2_ lipids in atomistic resolution.

### Quantification and statistical analysis

Statistical analyses were performed using GraphPad Prism 9.0. Two-tailed unpaired t-tests were performed. P< 0.05 was considered significant difference. P-value > 0.05 is indicated as no significant difference (ns). Data are plotted as mean ± S.D. from three independent experiments.

## Data Availability

• Data supporting the findings of this study have been deposited at Mendeley Data and are publicly available as of the date of publication. The DOI is listed in the key resources table. • This paper does not report original code. • Any additional information required to reanalyze the data reported in this paper is available from the lead contact upon request.

## Supplemental information

Document S1. Figures S1–S7 and Tables S1 and S2

Document S2. Article plus supplemental information
