# Supplementary material for: The solute carrier SPNS2 recruits PI(4,5)P2 to synergistically regulate transport of sphingosine-1-phosphate
Source: Mol Cell. 2023 Aug 3;83(15):2739–2752.e5. doi: 10.1016/j.molcel.2023.06.033 (PMC10790328; doi:10.1016/j.molcel.2023.06.033)
Supplement: Document S1. Figures S1–S7 and Tables S1 and S2 [file mmc1.pdf]

Molecular Cell, Volume *83*

Supplemental information

The solute carrier SPNS2 recruits PI(4,5)P_2 to synergistically regulate transport of sphingosine-1-phosphate

Haiping Tang, Huanyu Li, Dheeraj Prakaash, Conrado Pedebos, Xingyu Qiu, David B. Sauer, Syma Khalid, Katharina Duerr, and Carol V. Robinson

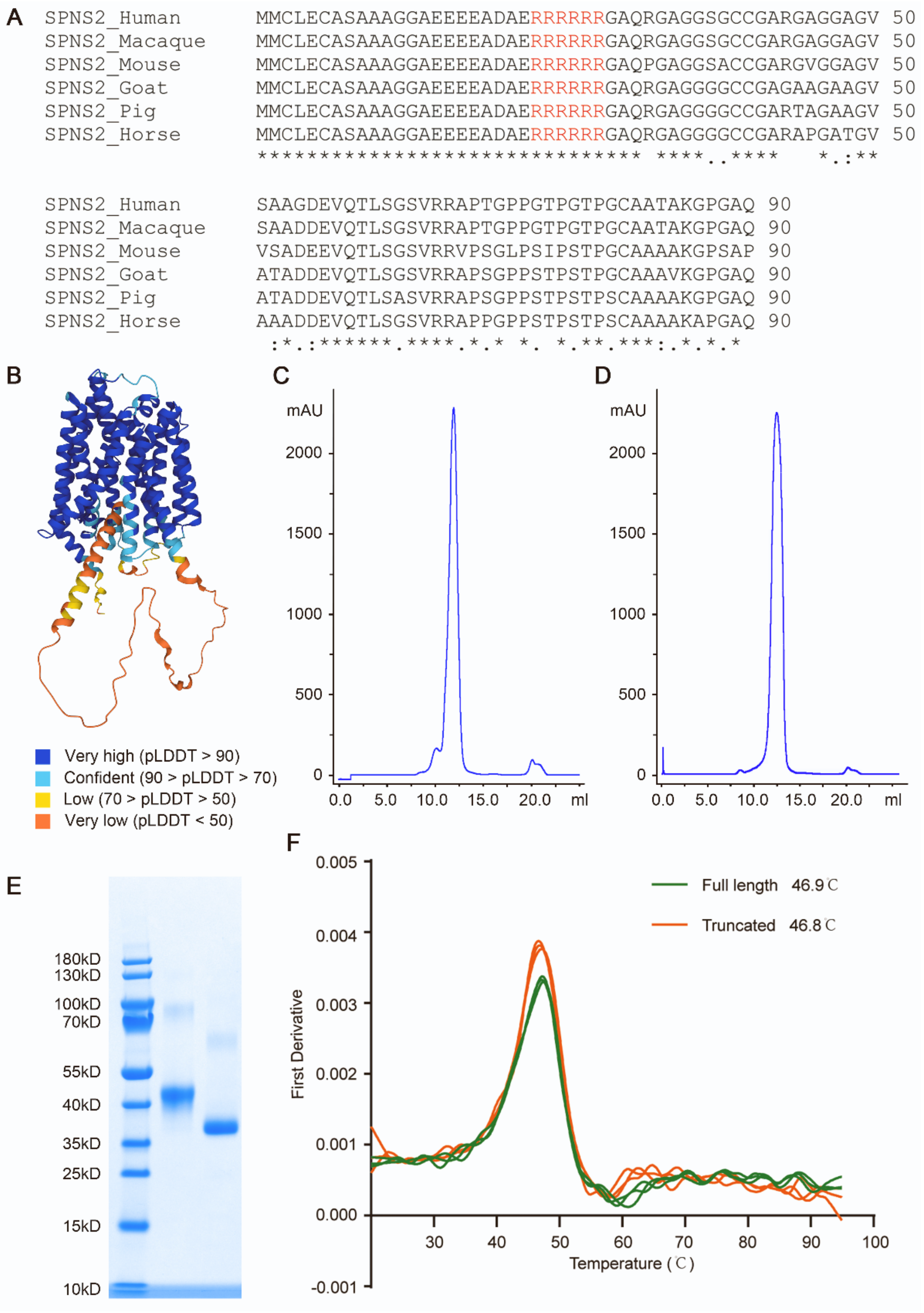

Figure S1. Amino acid sequence alignment of the N-terminus of SPNS2 orthologs and biochemical characterizations of SPNS2. Related to Figure 1.

(A) Amino acid sequence alignment of human SPNS2 (Uniprot ID Q8IVW8), macaque SPNS2 (Uniprot ID F6WND5), mouse SPNS2 (Uniprot ID Q91VM4), goat SPNS2 (Uniprot ID

A0A452G0V3), pig SPNS2 (Uniprot ID A0A286ZN28) and horse SPNS2(Uniprot ID F6X8L3). The sequence similarity is shown with asterisks (*) for identical residues and colon (:) for highly similar residues. Conserved residues forming the Arg patch are highlighted in red.
(B) AlphaFold2 predicted model of SPNS2 coloured with model confidence levels.
(C) Size-exclusion chromatography profile of full-length SPNS2.
(D) Size-exclusion chromatography profile of truncated SPNS2.
(E) Representative SDS-PAGE gel of purified SPNS2 proteins. Lane1, molecular weight maker; Lane 2, full-length SPNS2 protein; Lane 3, truncated SPNS2 protein.
(F) Nano-DSF melting curves of full-length and truncated SPNS2, indicating similar thermal stabilities.

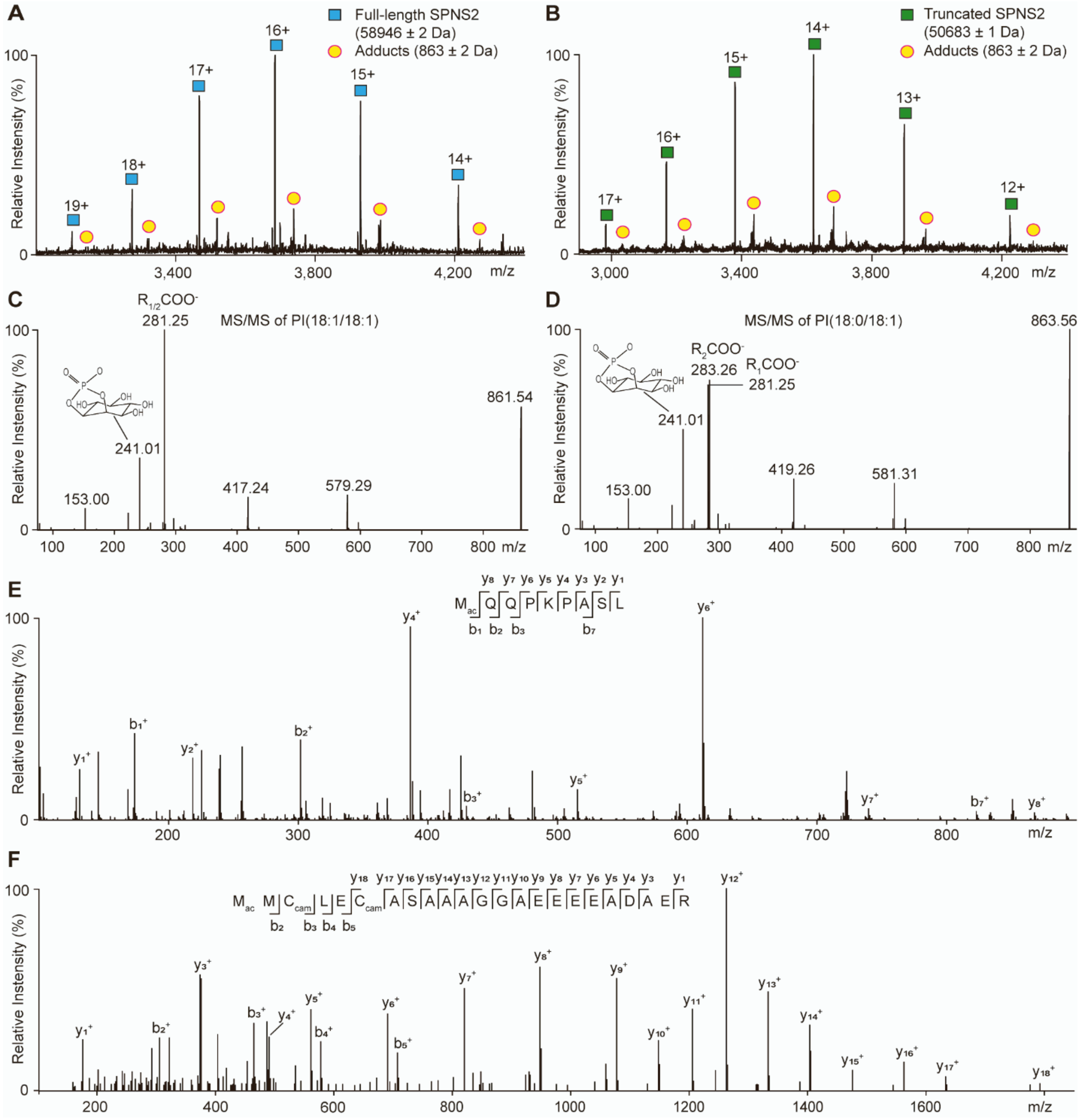

Figure S2. Identification of endogenous lipids bound to SPNS2 and N-terminal acetylation of truncated and full-length SPNS2. Related to Figure 1.

(A) Mass spectrum of full-length SPNS2 (blue, charge states 14+ to 19+ are shown) with bound adducts (mass of 863 ± 2 Da) (yellow circles).

(B) Mass spectrum of truncated SPNS2 (green, charge states 12+ to 17+) with bound adducts (mass of 863 ± 2 Da) (yellow circles).

Lipidomics analysis of purified full-length and truncated SPNS2 proteins reveals the identification of PI(18:1/18:1) and PI(18:0/18:1).

(C) MS/MS spectrum of PI(18:1/18:1).

(D) MS/MS spectrum of PI(18:0/18:1).
(E) MS/MS spectrum of peptide MQQPKPASL modified with acetylation (ac) at the methionine residues of truncated SPNS2 digested with chymotrypsin.
(F) MS/MS spectrum of peptide MMCLECASAAAGGAEEEEADAER modified with acetylation (ac) at the N-terminal methionine and cysteine carbamidomethylations (cam) of full-length SPNS2 digested with trypsin.

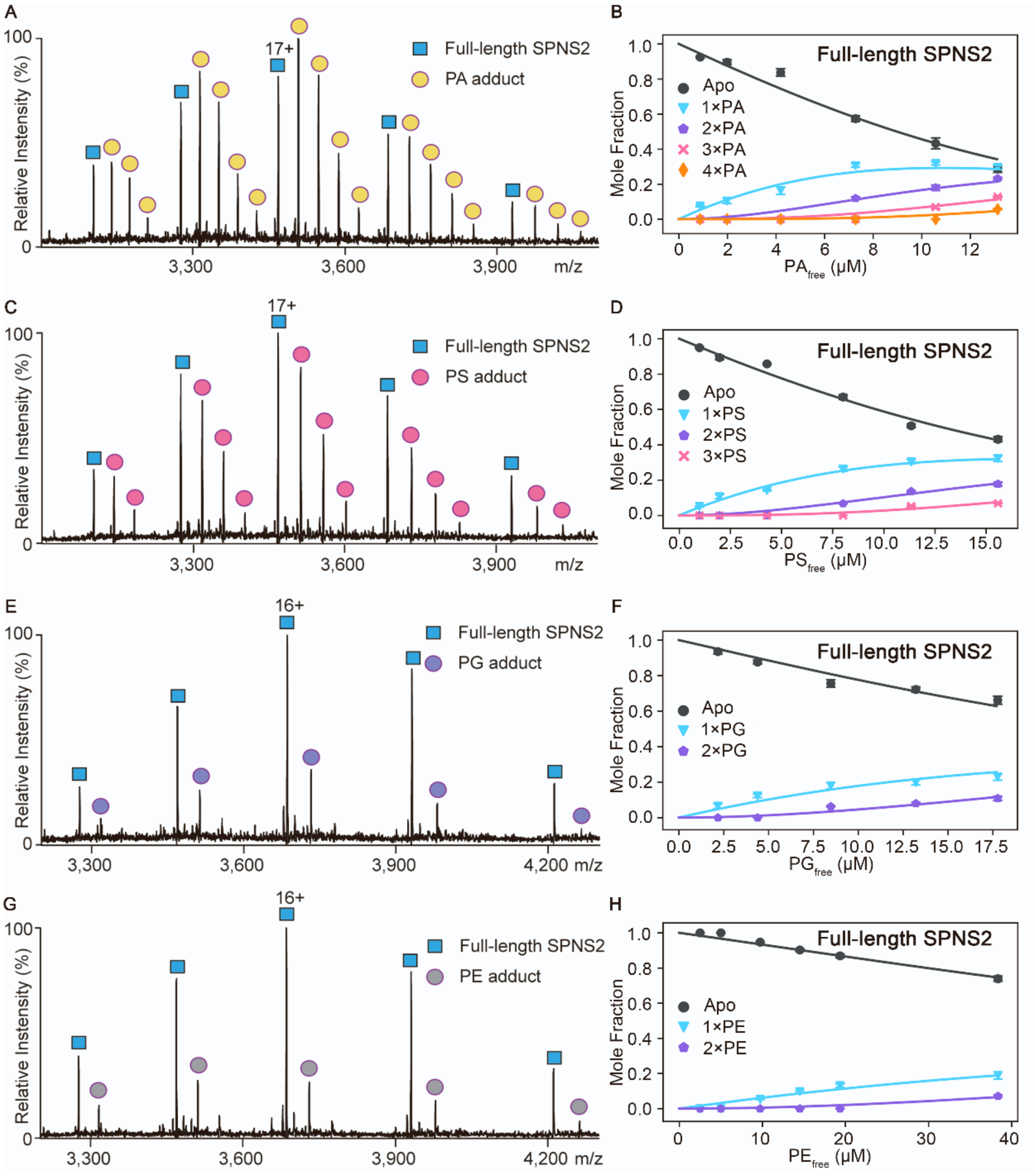

Figure S3. Determination of K_D values for phospholipids binding to full-length SPNS2. Related to Figures 1 and 2.

Shown are representative mass spectra for full-length SPNS2 in the presence of 20 µM of lipids except for PE (40 µM) (left panel). Plots of mole fraction (right panel) for *apo* full-length SPNS2 and bound to different number of lipids determined from a titration series (dots with error bars to represent the three measurements) and resulting fit from a sequential lipid-binding model (solid lines). All data are plotted as mean ± s.d.(n=3).

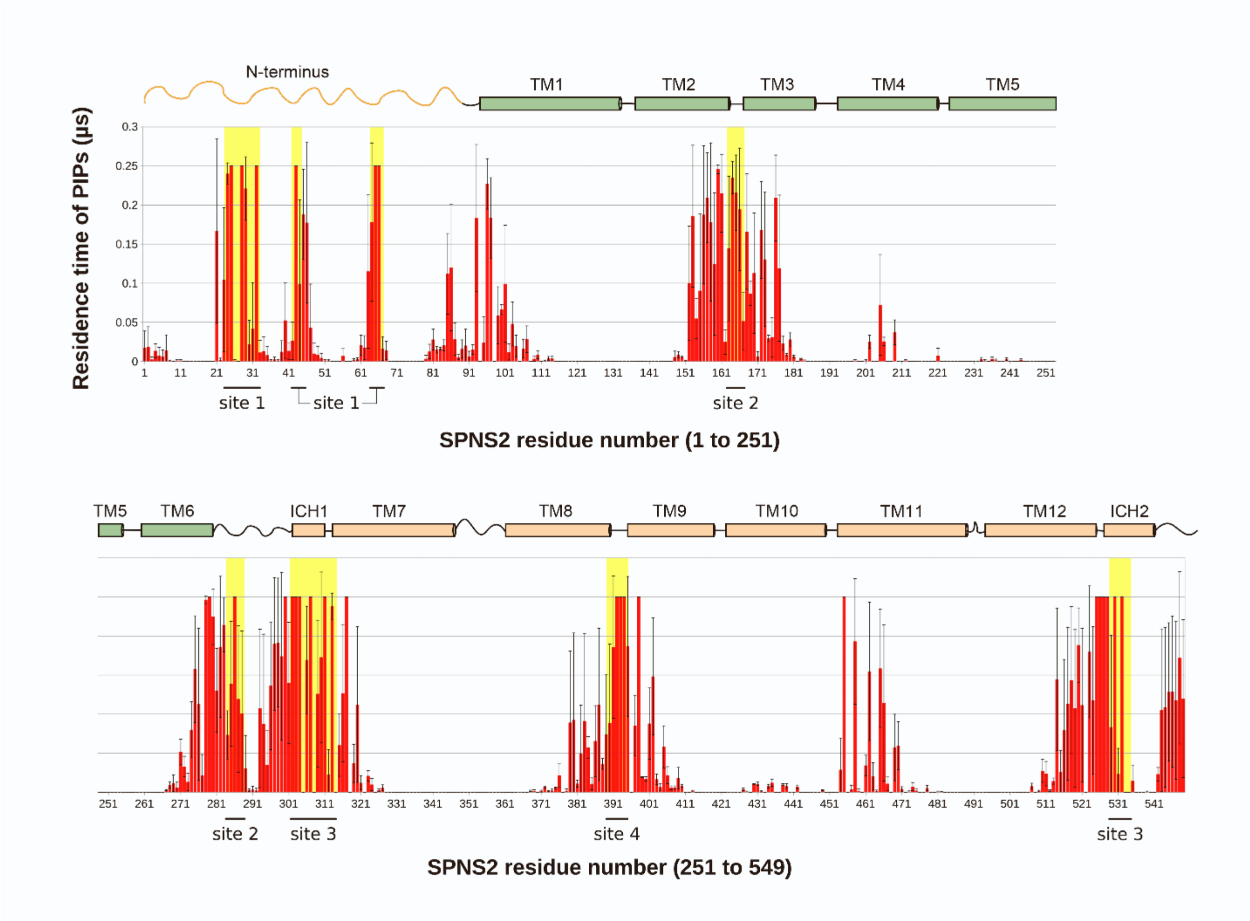

Figure S4. Residence times of PI(4,5)P_2 molecules interacting with SPNS2. Related to Figures 2 and 3.

The residence time histograms (in microseconds) were calculated from 3 independent equilibrium MD simulations and then averaged for each residue. Error bars for each residue represent standard deviations for their residence times. Each PI(4,5)P_2 binding site of SPNS2 contains positively charged residues that strongly interact with PI(4,5)P_2 lipids. These binding sites are labelled in the histograms. For clarity, histograms are split into two plots (SPNS2 residue numbers 1 to 251 - middle; residue numbers 251 to 549 - bottom).

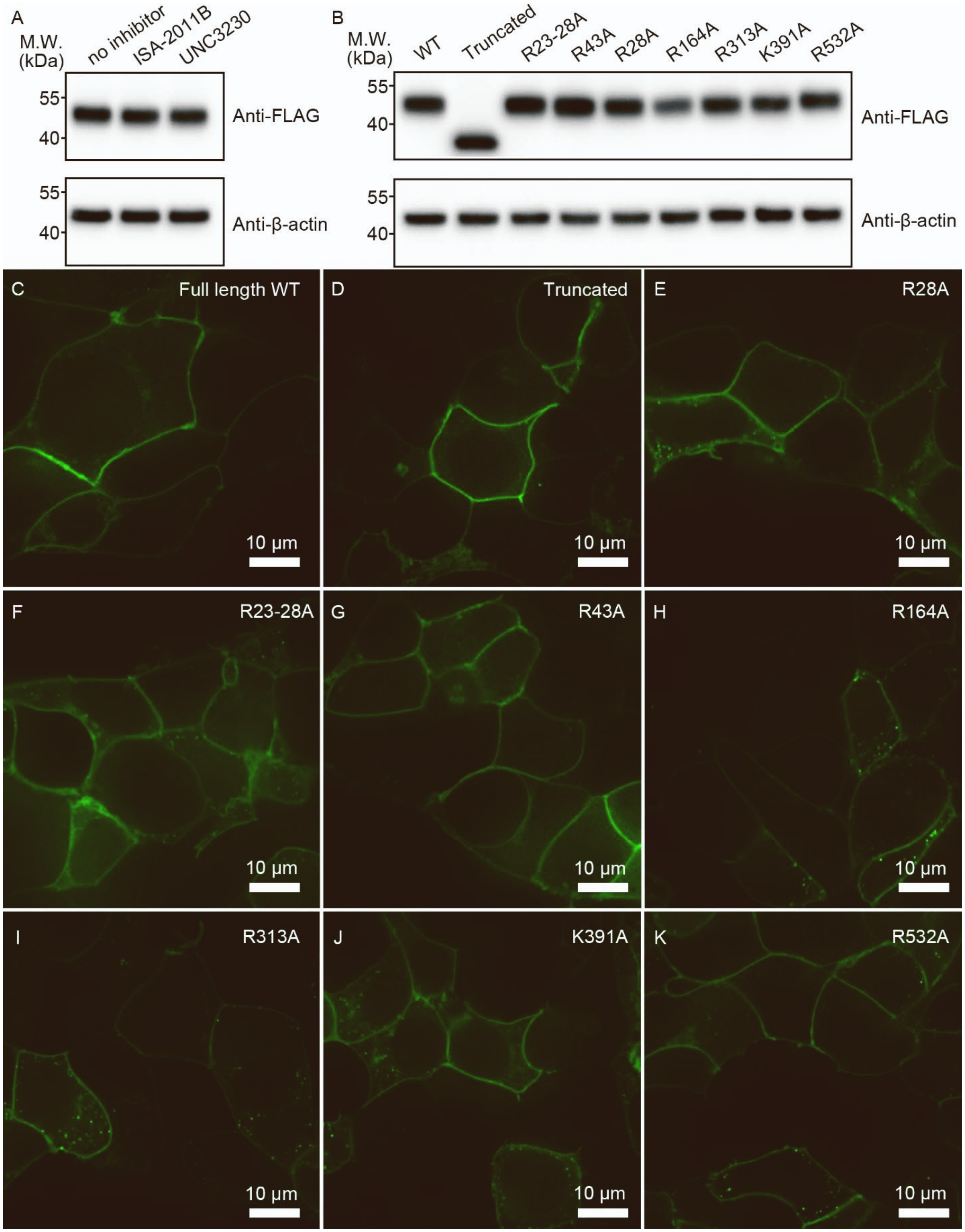

Figure S5. Western blot and confocal images of HEK293T cells expressing SPNS2 variants. Related to Figure 3.

(A) Western blot analysis of the expression levels of SPNS2 in SPNS2 overexpression cells in the absence and presence of PIP5K inhibitor ISA-2011B or UNC3230. β-actin was used as a loading control.

(B) Western blot analysis of the expression levels of SPNS2 variants. β-actin was used as a loading control.

(C) Confocal image of HEK293T cells expressing full-length WT SPNS2. Scale bars, 10 μm.

(D) Confocal image of HEK293T cells expressing truncated SPNS2. Scale bars, 10 μm.

(E) Confocal image of HEK293T cells expressing R28A substitution. Scale bars, 10 μm.

(F) Confocal image of HEK293T cells expressing R23-28A substitutions. Scale bars, 10 μm.

(G) Confocal image of HEK293T cells expressing R43A substitution. Scale bars, 10 μm.

(H) Confocal image of HEK293T cells expressing R164A substitution. Scale bars, 10 μm.

(I) Confocal image of HEK293T cells expressing R313A substitution. Scale bars, 10 μm.

(J) Confocal image of HEK293T cells expressing K391A substitution. Scale bars, 10 μm.

(K) Confocal image of HEK293T cells expressing R532A substitution. Scale bars, 10 μm.

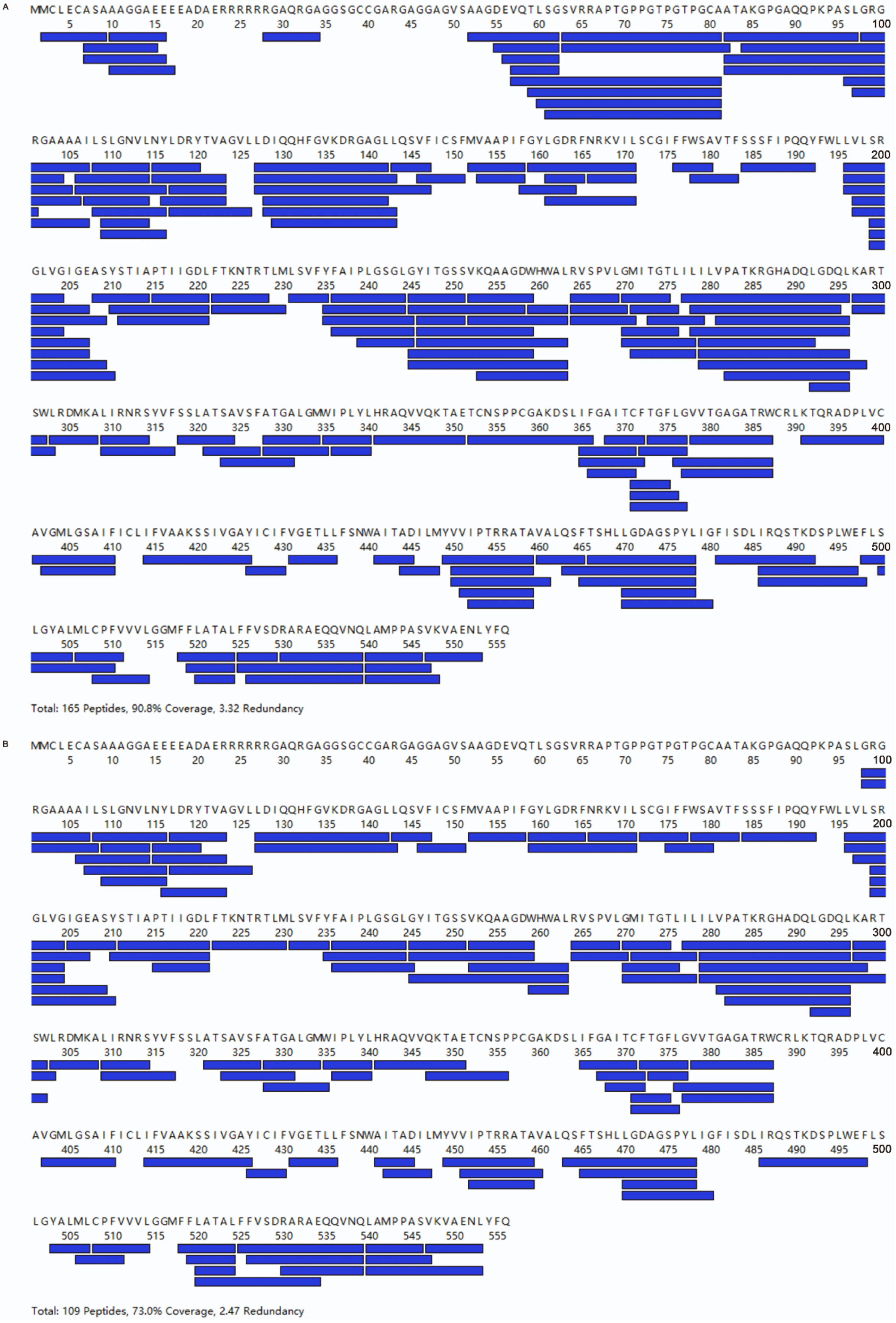
A
MMCLECASAAAGGAEEEEADAERRRRRRGAQRGAGGSGCCGARGAGGAGVSAAGDEVQTLSGSVRRAPTGPPGTPGTPGCAATAKGPGAQQPKPASLGRG
RGAAAAILSLGNVLNYLDRYTVAGVLLDIQQHFGVKDRGAGLLQSVFICSFMVAAPIFGYLGDRFNRKVILSCGIFFWSAVTFSSSFIPQQYFWLLVLSR
GLVGIGEASYSTIAPTIIGDLFTKNTRTLMLSVFYFAIPLGSGLGYITGSSVKQAAGDWHWALRVSPVLGMITGTLILILVPATKRGHADQLGDQLKART
SWLRDMKALIRNRSYVFSSLATSAVSFATGALGMWIPLYLHRAQVVQKTAETCNSPPCGAKDSLIFGAITCFTGFLGVVTGAGATRWCRLKTQRADPLVC
AVGMLGSAIFICLIFVAAKSSIVGAYICIFVGETLLFSNWAITADILMYVVIPTRRATAVALQSFTSHLLGDAGSPYLIGFISDLIRQSTKDSPLWEFLS
LGYALMLCPFVVVLGGMFFLATALFFVSDRARAEQQVNQLAMPPASVKVAENLYFQ
Total: 165 Peptides, 90.8% Coverage, 3.32 Redundancy
B
MMCLECASAAAGGAEEEEADAERRRRRRGAQRGAGGSGCCGARGAGGAGVSAAGDEVQTLSGSVRRAPTGPPGTPGTPGCAATAKGPGAQQPKPASLGRG
RGAAAAILSLGNVLNYLDRYTVAGVLLDIQQHFGVKDRGAGLLQSVFICSFMVAAPIFGYLGDRFNRKVILSCGIFFWSAVTFSSSFIPQQYFWLLVLSR
GLVGIGEASYSTIAPTIIGDLFTKNTRTLMLSVFYFAIPLGSGLGYITGSSVKQAAGDWHWALRVSPVLGMITGTLILILVPATKRGHADQLGDQLKART
SWLRDMKALIRNRSYVFSSLATSAVSFATGALGMWIPLYLHRAQVVQKTAETCNSPPCGAKDSLIFGAITCFTGFLGVVTGAGATRWCRLKTQRADPLVC
AVGMLGSAIFICLIFVAAKSSIVGAYICIFVGETLLFSNWAITADILMYVVIPTRRATAVALQSFTSHLLGDAGSPYLIGFISDLIRQSTKDSPLWEFLS
LGYALMLCPFVVVLGGMFFLATALFFVSDRARAEQQVNQLAMPPASVKVAENLYFQ
Total: 109 Peptides, 73.0% Coverage, 2.47 Redundancy

Figure S6. Sequence coverage of full-length and truncated SPNS2. Related to Figures 4, 5 and 6.

(A)165 peptides covering 90.8% of full-length SPNS2 sequence were identified following digestion with immobilized pepsin.

(B) 109 peptides covering 73% of truncated SPNS2 were identified following digestion with immobilized pepsin.

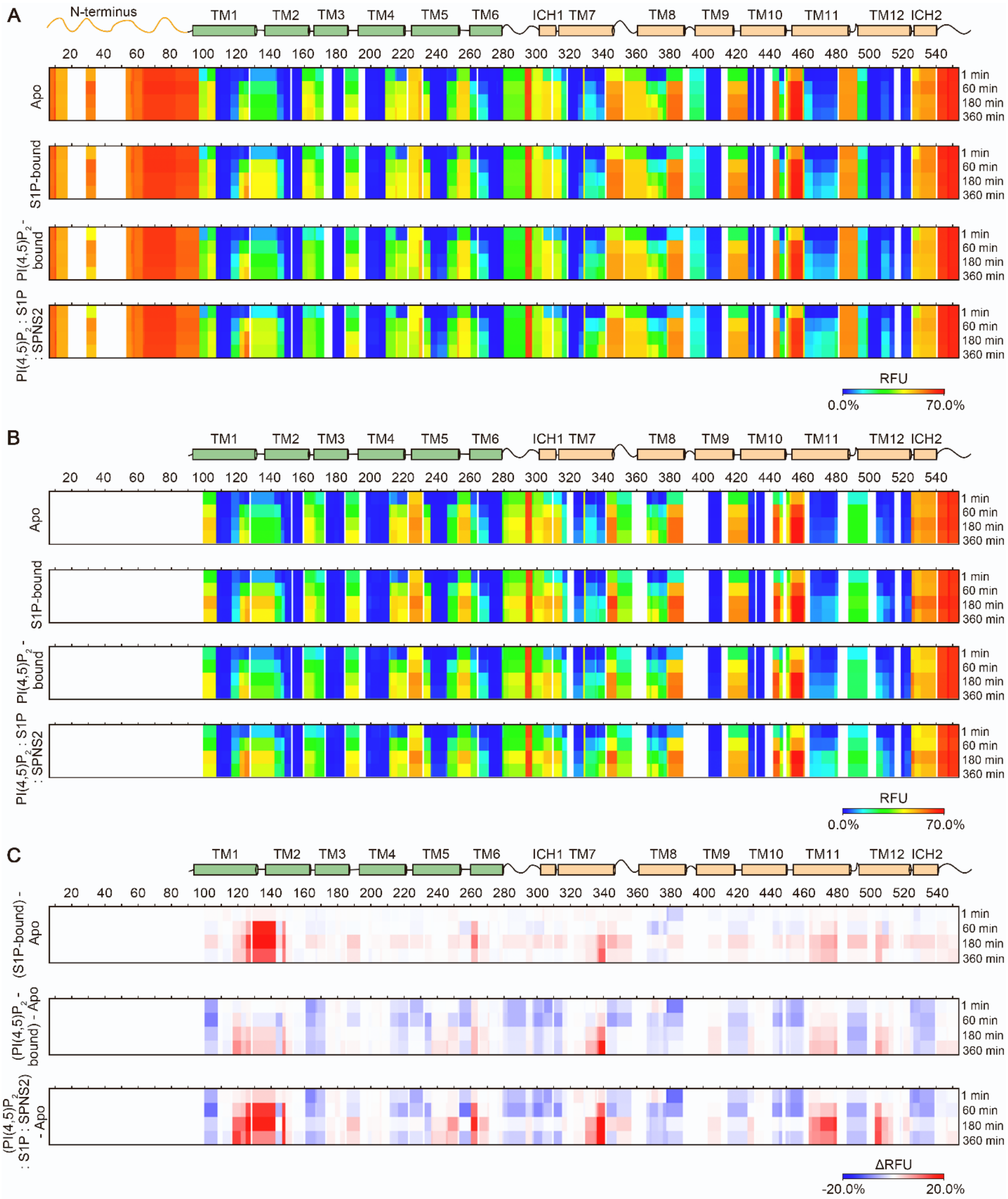

Figure S7. Heat map representing the RFU and ΔRFU of full-length and truncated SPNS2 in *apo* forms and upon S1P and PI(4,5)P_2 binding. Related to Figures 4, 5 and 6.

(A) Heatmap showing the RFU of full-length *apo* SPNS2, S1P-bound, PI(4,5)P_2-bound and ternary complex (PI(4,5)P_2: S1P: SPNS2) as a function of time.

(B) Heatmap showing the RFU of truncated *apo* SPNS2, S1P-bound, PI(4,5)P_2-bound and ternary complex (PI(4,5)P_2: S1P: SPNS2) as a function of time.

(C) Heatmap showing the time-dependent ΔRFU of truncated SPNS2, comparing S1P-bound SPNS2, PI(4,5)P_2-bound SPNS2 and ternary complex (PI(4,5)P_2: S1P: SPNS2) to *apo* SPNS2.

Table S1. K_D values for PI derivatives binding to full-length and truncated SPNS2 determined by native MS. Related to Figures 1 and 2.

Lipids	K_D	Full-length	Truncated
PI	K_{D1}	5.5 ± 0.2 μM	95 ± 4 μM
	K_{D2}	10.5 ± 0.4 μM	169 ± 10 μM
	K_{D3}	15.4 ± 0.8 μM	214 ± 16 μM
	K_{D4}	24.4 ± 1.4 μM	
PI(4)P	K_{D1}	6.4 ± 0.6 μM	31 ± 0.8 μM
	K_{D2}	9.9 ± 0.3 μM	83 ± 1 μM
	K_{D3}	13.7 ± 0.5 μM	132 ± 6 μM
	K_{D4}	20 ± 1 μM	147 ± 13 μM
$PI(4,5)P_2$	K_{D1}	0.7 ± 0.2 μM	9.9 ± 0.1 μM
	K_{D2}	3.0 ± 0.3 μM	30 ± 1μM
	K_{D3}	7.5 ± 1.3 μM	60 ± 3 μM
	K_{D4}	9.2 ± 1.2 μM	82 ± 5 μM
PIP_3	K_{D1}	6.3 ± 0.2 μM	14 ± 0.5 μM
	K_{D2}	13.3 ± 1.0 μM	50 ± 1 μM
	K_{D3}	20.8 ± 0.5 μM	87 ± 1 μM
	K_{D4}	38.1 ± 2.7 μM	125 ± 7 μM

Table S2. K_D values for glycerophospholipids binding to full-length SPNS2 determined by native MS. Related to Figures 1 and 2.

Lipids	K_D	Full length SPNS2
PA	K_{D1}	15.7 ± 0.9 μM
	K_{D2}	17.5 ± 0.4 μM
	K_{D3}	25.0 ± 0.9 μM
	K_{D4}	31.7 ± 2.3 μM
PS	K_{D1}	20.8 ± 0.6 μM
	K_{D2}	27.5 ± 1.7 μM
	K_{D3}	37.5 ± 1.3 μM
PG	K_{D1}	43.6 ± 2.7 μM
	K_{D2}	39.4 ± 3.6 μM
PE	K_{D1}	152.0 ± 10.1 μM
	K_{D2}	111.0 ± 11.8 μM
